# Unsupervised learning of stationary and switching dynamical system models from Poisson observations

**DOI:** 10.1088/1741-2552/ad038d

**Published:** 2023-12-12

**Authors:** Christian Y Song, Maryam M Shanechi

**Affiliations:** 1 Ming Hsieh Department of Electrical and Computer Engineering, Viterbi School of Engineering, University of Southern California, Los Angeles, CA, United States of America; 2 Neuroscience Graduate Program, University of Southern California, Los Angeles, CA, United States of America; 3 Alfred E. Mann Department of Biomedical Engineering, Viterbi School of Engineering, University of Southern California, Los Angeles, CA, United States of America; 4 Thomas Lord Department of Computer Science, Viterbi School of Engineering, University of Southern California, Los Angeles, CA, United States of America

**Keywords:** Poisson observations, unsupervised learning, dynamical system models, deterministic sampling, switching state space

## Abstract

*Objective*. Investigating neural population dynamics underlying behavior requires learning accurate models of the recorded spiking activity, which can be modeled with a Poisson observation distribution. Switching dynamical system models can offer both explanatory power and interpretability by piecing together successive regimes of simpler dynamics to capture more complex ones. However, in many cases, reliable regime labels are not available, thus demanding accurate unsupervised learning methods for Poisson observations. Existing learning methods, however, rely on inference of latent states in neural activity using the Laplace approximation, which may not capture the broader properties of densities and may lead to inaccurate learning. Thus, there is a need for new inference methods that can enable accurate model learning. *Approach*. To achieve accurate model learning, we derive a novel inference method based on deterministic sampling for Poisson observations called the Poisson Cubature Filter (PCF) and embed it in an unsupervised learning framework. This method takes a minimum mean squared error approach to estimation. Terms that are difficult to find analytically for Poisson observations are approximated in a novel way with deterministic sampling based on numerical integration and cubature rules. *Main results*. PCF enabled accurate unsupervised learning in both stationary and switching dynamical systems and largely outperformed prior Laplace approximation-based learning methods in both simulations and motor cortical spiking data recorded during a reaching task. These improvements were larger for smaller data sizes, showing that PCF-based learning was more data efficient and enabled more reliable regime identification. In experimental data and unsupervised with respect to behavior, PCF-based learning uncovered interpretable behavior-relevant regimes unlike prior learning methods. *Significance*. The developed unsupervised learning methods for switching dynamical systems can accurately uncover latent regimes and states in population spiking activity, with important applications in both basic neuroscience and neurotechnology.

## 1. Introduction

Investigating neural dynamics underpinning naturalistic behaviors requires models of neural population activity that can balance explanatory power and interpretability. One class of models that can offer such a balance is a switching dynamical system model [1–4]. These switching models often use linear dynamics to describe the time evolution of neural population activity in terms of an underlying latent state; however, they allow for a switch non-stationarity such that a series of distinct linear dynamics called regimes can be pieced together to capture more complex neural dynamics. The ability of these models to capture switch non-stationarity could enable investigations of complex movement patterns and switching tasks [4–7], switches in attention and decision-making strategies [3, 8, 9], and even switches in how behavior is encoded in neural activity [10]. However, learning accurate switching models based on neural population activity data is a necessity for such endeavors, especially in the absence of reliable trial labels and/or regime labels.

Neural population activity is typically measured in the form of spiking activity [11]. The binary nature of a spike event [12–18] can introduce challenges in the inference stage of various learning frameworks aimed at training data-driven models. We define inference as using observed neural activity to estimate latent states that give rise to both behavior and neural activity [11, 19–21] given model parameters. Prior learning methods for both stationary and switching dynamical systems that model spikes as discrete Poisson observations have used the Laplace approximation for inference [3, 12, 14, 22–25]. However, this approximation has the potential to lead to inaccurate learning as we will also show in our Results since it may not be able to capture the broader properties of the target of approximation [26]. Thus, there is a need for more accurate learning methods that can better learn the model parameters for both stationary and switching dynamical system models, and in turn facilitate the investigations of neural dynamics underlying behavior.

For unsupervised learning, expectation–maximization (EM) is a commonly used two-step iterative framework. EM finds the model parameters according to a cost function that maximizes the log-likelihood of the observed neural activity in the training data [27], without any information about the latent states or regimes. The two steps are the expectation step (E-step) and the maximization step (M-step), where the E-step involves the inference of unobserved latent states. For switching systems, such exact inference is inherently intractable regardless of the observation modality [2], thus requiring an additional layer of approximation. Prior work called variational Laplace-EM (vLEM) [3] has made great progress using a mean-field variational approximation [28, 29] in a variational EM framework. However, variational EM has to modify the cost function to maximize the lower bound to the log-likelihood rather than the log-likelihood itself [29–31]. More importantly, in the E-step, vLEM also uses the Laplace approximation, which has the potential to lead to inaccurate learning.

An alternative to a variational EM framework is what we will call a switch EM framework. The switch EM framework uses switching filters and smoothers [1, 2, 32–34] in the E-step to directly maximize the log-likelihood rather than its lower-bound as is done in variational EM. Switch EM has been successful for continuous Gaussian observations [6, 35] because accurate stationary filters can be incorporated in the switching filter in this case, specifically the Kalman filter which is optimal for Gaussian observations [30, 36]. Recent work [1] has extended switching filters to allow for Poisson observations using the Laplace approximation in the spirit of prior point process filters (PPF) [13, 16, 17, 22–24]. However, because the switch filtering method in this work [1] relies on incorporating stationary filters that are Laplace approximation-based, it does not have sufficient accuracy for model learning as we will show in our Results. Thus, there is a need for a more accurate filter to enable a successful switch EM framework for Poisson observations.

**Figure 1.**
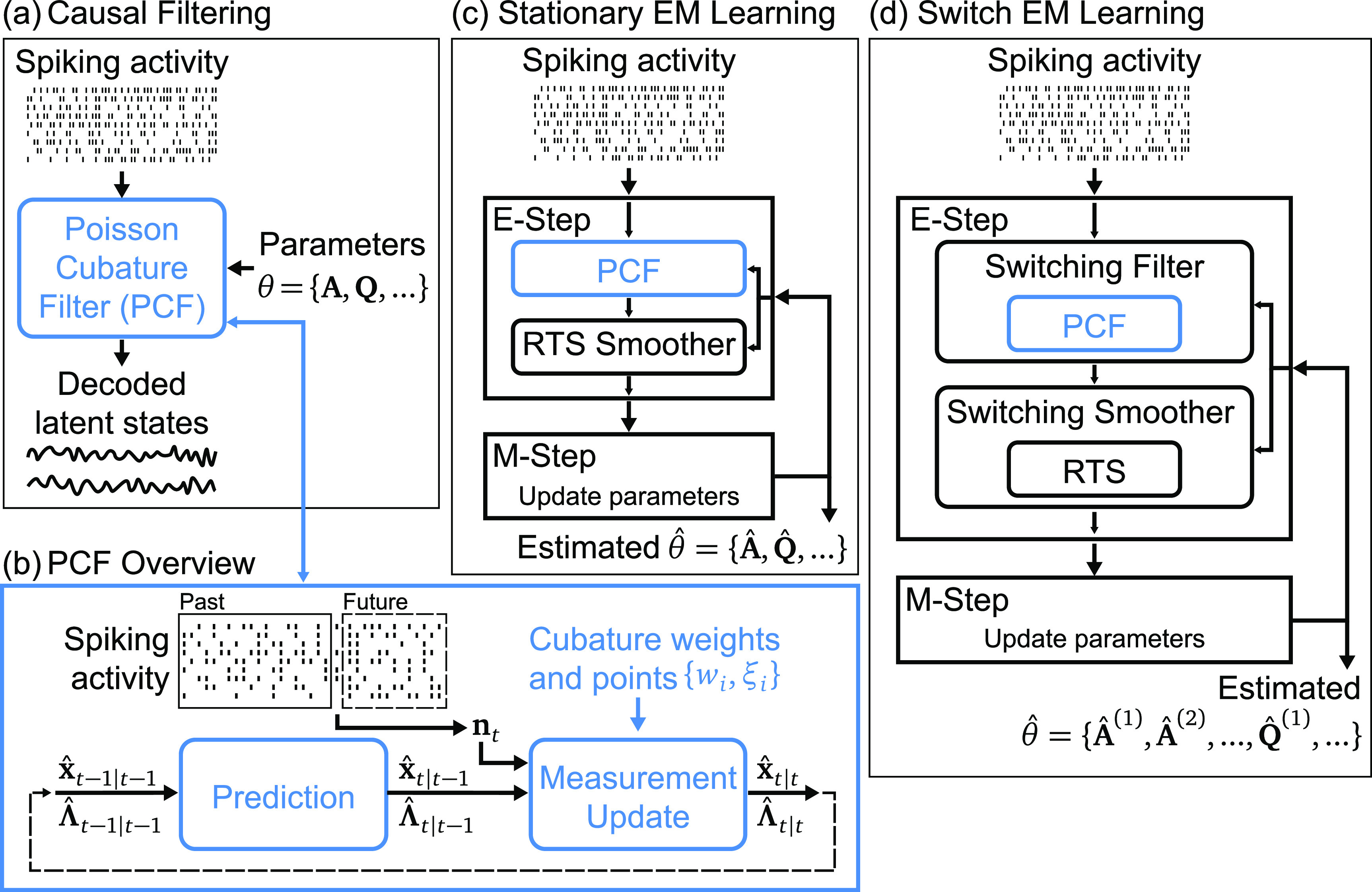
Poisson Cubature Filter (PCF) and how it enables EM learning for stationary and switching systems with Poisson observations. (a) PCF takes spiking activity modeled as Poisson observations and model parameters to causally decode unobserved latent states. (b) PCF is a recursive filter that is comprised of a prediction step followed by a measurement update step that is performed by utilizing deterministically sampled points based on cubature rules from numerical integration techniques. (c) PCF is embedded in an EM framework to enable accurate unsupervised learning of model parameters using only spiking activity as input. It is paired with an RTS smoother in the E-Step of EM. (d) PCF is also embedded in a switch EM framework to enable unsupervised learning of parameters for a switching dynamical system with Poisson observations. It is first embedded within a switching filter and then paired with a switching smoother to comprise the E-Step of switch EM.

With the goal to enable accurate unsupervised learning of both stationary and switching dynamical systems with Poisson observations, here we develop a novel deterministic sampling filter for Poisson observations called the Poisson Cubature Filter (PCF). The development of PCF for learning was motivated by the empirical results using a Laplace approximation-based filter in EM learning frameworks, which suggested the need for a more accurate filter for learning purposes. Prior deterministic sampling filters [37–40] however use a specific non-linear observation formulation that is not applicable to Poisson observations, thus necessitating a new filter. We start the development of PCF from a minimum mean-squared error (MMSE) approach to estimation which consists of various MMSE terms. However, these necessary MMSE terms are difficult to calculate analytically for Poisson observations. PCF overcomes this challenge by converting these terms using novel derivations into a form that enables the use of a numerical integration tool to approximate the terms. We then embed PCF in both stationary and switch EM frameworks as overviewed in figure 1 to enable accurate unsupervised learning of model parameters from just spiking activity observations.

We first validate our methods with numerical simulations. We show that EM frameworks with PCF successfully learn accurate model parameters for both stationary and switching systems. Compared with learning methods that use the Laplace approximation for inference, the new method can learn the parameters significantly more accurately and improve the neural self-prediction, behavior decoding, and regime decoding. Also, comparisons with the same switch EM framework that instead uses the Laplace approximation-based PPF shows that successful learning depends on using the new PCF. We finally show that the benefits of switch EM with PCF are greater with smaller data sizes, showing that the method is more data-efficient and more reliable in identifying regimes in data compared to prior learning methods.

**Figure 2.**
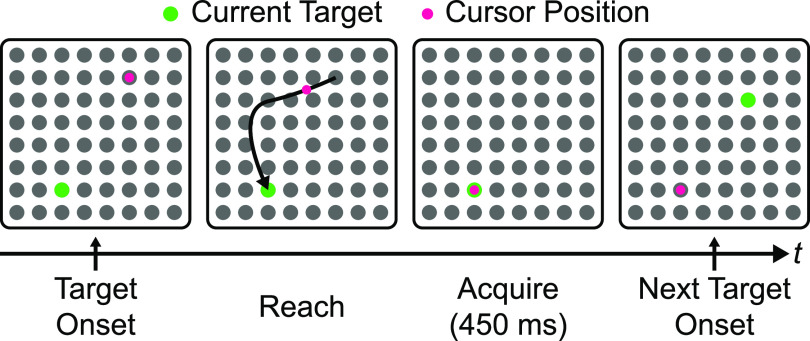
Behavioral task overview. The task is a point-to-point task consisting of a continuous series of reaches to targets. To acquire the current target, the subject must hold on the target for 450 ms. Upon doing so, a new random target from the 8-by-8 grid is then presented, thus starting the next trial. The task is continuous and contains no pre-movement delay periods.

We then show the success of both stationary and switch EM learning with PCF in publicly available motor cortical spiking activity data acquired from [41] from a non-human primate performing a continuous point-to-point reach task overviewed in figure 2. We find that the new PCF-based learning method for switching models succeeds in tracking regimes as evident by its significantly better decoding metrics compared to learned stationary models. We also find that PCF-based learning methods can outperform Laplace approximation-based methods in terms of the neural self-prediction and behavior decoding ability of learned parameters for both stationary and switching dynamical system models. Interestingly, despite being fully unsupervised with respect to behavior, we find that PCF-based learning uncovers interpretable and behavior-relevant regimes unlike prior learning methods. These results show the potential of the PCF-based EM frameworks for learning stationary and switching dynamical system models from neural datasets.

## 2. Results

We first provide a brief overview of the model and methods with full details available in section 4. We then show in both numerical simulations and in neural spiking activity recorded from primary motor cortex during behavior that unsupervised EM learning methods embedded with the developed PCF successfully learn system parameters and outperform prior methods for both stationary and switching systems. We further show that the PCF-based switch learning better identifies regimes in both simulated and experimental data and is more sample/data efficient. We use the Wilcoxon signed-rank test for all statistical comparisons and control for false discovery rate using the Benjamini–Hochberg Procedure [42].

### 2.1. Overview of unsupervised EM learning with PCF

We use a switching dynamical system model with Poisson observations:

\begin{align*}\begin{array}{*{20}{l}} {P\left( {s_t^{\left(\, j \right)}|s_{t - 1}^{\left( i \right)}} \right) = {{{\Phi }}_{j,i}}} \\[3pt] {{{\mathbf{x}}_t} = {\mathbf{A}}\left( {{s_t}} \right){{\mathbf{x}}_{t - 1}} + {{\mathbf{w}}_t},{{\mathbf{w}}_t} \sim N\left( {0,{\mathbf{Q}}\left( {{s_t}} \right)} \right)} \\[3pt] {P\left( {{{\mathbf{n}}_t}|{{\mathbf{x}}_t},{s_t}} \right) = \mathop \prod \limits_{i = 1}^C \frac{{{{\left( {{\lambda _i}\left( {{{\mathbf{x}}_t},{s_t}} \right){{\Delta }}} \right)}^{n_t^i}}\exp \left( { - {\lambda _i}\left( {{{\mathbf{x}}_t},{s_t}} \right){{\Delta }}} \right)}}{{n_t^i!}}{\text{ }}} \\[3pt] {{\lambda _i}\left( {{{\mathbf{x}}_t},{s_t}} \right){{\Delta }} = \exp \left( {{\alpha _i}\left( {{s_t}} \right) + {{\boldsymbol{\unicode{x03B2}}}_i}{{\left( {{s_t}} \right)}^T}{{\mathbf{x}}_t}} \right)} \end{array}.\end{align*}

Here, the latent brain state ${{\mathbf{x}}_t} \in {\mathbb{R}^d}$ evolves with linear dynamics dictated by ${\mathbf{A}}\left( {{s_t}} \right)$ and its eigenvalues. For the spiking activity, the latent state is encoded in the instantaneous firing rate of Poisson observations ${{\mathbf{n}}_t} = {\left[ {n_t^1, \ldots ,{\text{ }}n_t^C} \right]^T}$ through a log-link function. The regime state ${s_t} \in \left[ {1,M} \right]$ is a first-order Markov chain which dictates the parameters used throughout the model and enables the model’s functionality to describe non-stationary switches in dynamics and/or encoding. Stationary systems are then a special case of (1) with the number of regimes $M$ equal to 1. See sections 4.2 and 4.5.1 for further details.

For causal filtering with Poisson observations, the Laplace approximation is a popular approach, which is the basis for the prior PPF and can operate on Poisson observations [13, 16, 17, 22–24]. However, we find empirically that these methods can lead to inaccurate learning, especially in switching systems when the PPF is used in EM learning as we show in our below Results. We hypothesize that this inaccurate learning may be due to the inaccuracies in the Laplace approximation not being able to accurately capture first and second moments of densities that are being approximated [26]. More specifically, Laplace approximation may misrepresent first and second moments as shown in [26, 30, 43] for example in the case of skewed densities or densities in which the curvature at the mode that approximates the second-moment does not represent the mass in the tails. These issues may lead to inaccuracy in learning applications as second moment terms are critical in estimating system parameters (see section 4.4 and equation (44)). The approach of Laplace approximation-based filters resembles the non-linear filtering approach of the extended Kalman filter (EKF) [30] in that both of these approaches base their approximations around a single point through a Taylor series expansion [26]. While for continuous observations there are more accurate filters than EKF such as the unscented Kalman filter (UKF) and cubature Kalman filter (CKF) [30, 37–39, 44, 45] with comparable computational costs [30], these filters cannot be applied to Poisson observations. This is because these traditional non-linear filters generally assume the observation equation is of the form ${{\mathbf{n}}_t} = h\left( {{{\mathbf{x}}_t}} \right) + {{\mathbf{v}}_t}$ where $h\left( \cdot \right)$ non-linearly but deterministically transforms the latent state and ${{\mathbf{v}}_t}$ is some additive noise. However, for Poisson and other discrete observation modalities, the latent state is encoded in a probability distribution as in (1) with the actual observation being randomly drawn from that distribution. In other words, these modalities cannot be written in the above form, i.e., $h\left( \cdot \right)$ does not exist for Poisson nor is there an easy modification to directly allow for Poisson. Therefore, we need to derive a new filter starting from fundamental estimation principles to allow for more accurate unsupervised learning with Poisson observations.

Motivated by this need, we develop the PCF for unsupervised learning with EM. PCF is a recursive causal inference method for Poisson observations. We denote the estimation of latent state ${{\mathbf{x}}_t}$ given observations ${{\mathbf{n}}_{1:\tau }}$ as ${\hat {\mathbf{x}}_{t|\tau }}$ with estimated state covariance similarly denoted as ${\hat {\boldsymbol{\Lambda }}_{t|\tau }}$. As overviewed by figure 1(b), we start with the state estimate at the previous timestep ${\hat {\mathbf{x}}_{t - 1|t - 1}}$ and ${\hat {\boldsymbol{\Lambda }}_{t - 1|t - 1}}$ and perform a prediction step using the linear dynamics of the model similar to Kalman prediction to yield the predicted state ${\hat {\mathbf{x}}_{t|t - 1}}$ and ${\hat {\boldsymbol{\Lambda }}_{t|t - 1}}$ which is assumed to be Gaussian—this is a prediction because observations up to the previous timestep are used to predict the current state. The challenge comes in the following measurement update step that incorporates the new observation ${{\mathbf{n}}_t}$ to yield the state estimate at the present timestep ${\hat {\mathbf{x}}_{t|t}}$ and ${\hat {\boldsymbol{\Lambda }}_{t|t}}$.

The Poisson observation is nonconjugate with the Gaussian density of ${\hat {\mathbf{x}}_{t|t - 1}}$, thus requiring approximation. Prior work has used the approach of approximating the update density through the Laplace approximation to yield the PPF [13, 16, 17] which can operate on Poisson observations. However, given the potential inaccuracy of the Laplace approximation for unsupervised learning purposes, we take a different approach and aim to minimize the mean-squared error of a state estimator that has the form:

\begin{align*}\begin{aligned} {\hat {\mathbf{x}}_{t|t}} &amp; = {\hat {\mathbf{x}}_{t|t - 1}} + {{\boldsymbol{\Lambda }}_{{\mathbf{xn}}}}{\boldsymbol{\Lambda }}_{{\mathbf{nn}}}^{ - 1}\left( {{{\mathbf{n}}_t} - {{\hat {\mathbf{n}}}_{{\mathbf{t}}|{\mathbf{t}} - 1}}} \right) \hfill \\ {\hat {\boldsymbol{\Lambda }}_{t|t}} &amp; = {\hat {\boldsymbol{\Lambda }}_{t|t - 1}} - {{\boldsymbol{\Lambda }}_{{\mathbf{xn}}}}{\boldsymbol{\Lambda }}_{{\mathbf{nn}}}^{ - 1}{\boldsymbol{\Lambda }}_{{\mathbf{xn}}}^T \hfill \\ \end{aligned} .\end{align*}

For the above estimator form, the below terms are known to minimize the mean-squared error regardless of observation modality (see section 4.3).

\begin{align*}\begin{aligned} {\hat {\mathbf{n}}_{t|t - 1}} &amp; = E\left[ {{{\mathbf{n}}_t}{\text{|}}{{\mathbf{n}}_{1:t - 1}}} \right] \hfill \\ {{\boldsymbol{\Lambda }}_{{\mathbf{nn}}}} &amp; = V\left[ {{{\mathbf{n}}_t}{\text{|}}{{\mathbf{n}}_{1:t - 1}}} \right] \hfill \\ &amp; = E\left[ {\left( {{{\mathbf{n}}_t} - {{\hat {\mathbf{n}}}_{t|t - 1}}} \right){{\left( {{{\mathbf{n}}_t} - {{\hat {\mathbf{n}}}_{t|t - 1}}} \right)}^T}\left| {{{\mathbf{n}}_{1:t - 1}}} \right.} \right] \hfill \\ {{\boldsymbol{\Lambda }}_{{\mathbf{xn}}}} &amp; = E\left[ {\left( {{{\mathbf{x}}_t} - {{\hat {\mathbf{x}}}_{t|t - 1}}} \right){{\left( {{{\mathbf{n}}_t} - {{\hat {\mathbf{n}}}_{t|t - 1}}} \right)}^T}\left| {{{\mathbf{n}}_{1:t - 1}}} \right.} \right] \hfill \\ \end{aligned} .\end{align*}

These terms are expected values with respect to the non-Gaussian distributions $P\left( {{{\mathbf{n}}_t}|{{\mathbf{n}}_{1:t - 1}}} \right)$ and $f\left( {{{\mathbf{x}}_t},{{\mathbf{n}}_t}|{{\mathbf{n}}_{1:t - 1}}} \right)$ and are difficult to calculate analytically, necessitating approximation. One way for approximating these terms is numerical integration. Numerical integration can be used to approximate expected value terms if they are with respect to a Gaussian through a series of weighted sums at deterministically sampled points (see section 4.1). However, our expected values above are not with respect to Gaussians. Thus, to derive the new PCF with Poisson observations, we must overcome the challenge of analytically converting the expected value terms in (3) into expected values with respect to Gaussians first, so that we can then enable the use of numerical integration. See section 4.1 for an overview of numerical integration and section 4.3 for a detailed derivation of how we do so. We now present the final equations. The prediction step is as follows:

\begin{align*}\begin{array}{*{20}{l}} {{{\hat {\mathbf{x}}}_{t|t - 1}} = {\mathbf{A}}{{\hat {\mathbf{x}}}_{t - 1|t - 1}}} \\ {{{\hat {\boldsymbol{\Lambda }}}_{t|t - 1}} = {\text{ }}{\mathbf{A}}{{\hat {\boldsymbol{\Lambda }}}_{t - 1|t - 1}}{{\mathbf{A}}^T} + {\mathbf{Q}}} \end{array}.\end{align*}

The update step then updates the estimate with the current observation. The moment terms for the measurement update step are approximated with numerical integration as follows:

\begin{align*} {{\hat {\mathbf{n}}}_{t|t - 1}} &amp; \approx \mathop \sum \limits_{i = 1}^{2{d^2} + 1} {w_i} \cdot p\left( {{{\mathbf{x}}_i}} \right) \nonumber\\ {{\boldsymbol{\Lambda }}_{{\mathbf{nn}}}} &amp; \approx \mathop \sum \limits_{i = 1}^{2{d^2} + 1} {w_i} \cdot \left( {q\left( {{{\mathbf{x}}_i}} \right) + p\left( {{{\mathbf{x}}_i}} \right)p{{\left( {{{\mathbf{x}}_i}} \right)}^T}} \right)\nonumber\\ &amp; \quad - {{\hat {\mathbf{n}}}_{t|t - 1}}\hat {\mathbf{n}}_{t|t - 1}^T \nonumber\\ {\text{ }}{{\boldsymbol{\Lambda }}_{{\mathbf{xn}}}} &amp; \approx \mathop \sum \limits_{i = 1}^{2{d^2} + 1} {w_i} \cdot {{\mathbf{x}}_i}p{{\left( {{{\mathbf{x}}_i}} \right)}^T} - {{\hat {\mathbf{x}}}_{t|t - 1}}\hat {\mathbf{n}}_{t|t - 1}^T \nonumber\\ {{\mathbf{x}}_i} &amp; \triangleq {{\hat {\mathbf{x}}}_{t|t - 1}} + \sqrt {{{\hat {\boldsymbol{\Lambda }}}_{t|t - 1}}} {{\boldsymbol{\unicode{x03BE}}}_i} \end{align*}

where $p\left( \cdot \right)$ and $q\left( \cdot \right)$ are the functions for the likelihood mean and variance of Poisson given by (14) and $\left\{ {{w_i},{{\boldsymbol{\unicode{x03BE}}}_i}} \right\}$ are weights and points which are determined by a spherical–radial cubature rule given in (6) in section 4.1. Note that while Laplace approximation-based filters estimate the mean and covariance based on a single point in the latent space, PCF’s deterministic sampling method samples multiple points from the latent space.

Having derived the novel PCF, we then embed it in EM frameworks to yield unsupervised learning methods for both stationary and switching systems that estimate model parameters given only Poisson observations as overviewed by figures 1(c) and (d). *For stationary systems*, we use PCF with an Rauch–Tung–Striebel (RTS) smoother in the E-step which we refer to as PCF-EM. We then compare to existing stationary methods: (1) PPF-EM which in its EM uses the widely-used PPF with Laplace’s approximation [16, 17, 22–24] and (2) Laplace-EM (LEM) which uses the global Laplace method for the entire E-step where Laplace approximation is applied non-causally to the entire observation timeseries [12, 14, 25] with details in section 4.4. *For switching systems,* we use what we call a switch EM framework where the E-step is comprised of a bank of parallel PCF filters in a switching filter. We call this switching filter switching PCF and call the entire learning method sPCF-EM. We compare to the prior Variational Laplace-EM (vLEM) method [3] which uses a distinct variational EM framework [28, 29] with global Laplace embedded in the E-step. We emphasize that a successful switch EM framework that uses the prior PPF filters has not been shown before. Nevertheless, to show that PCF enables a more accurate and reliable switch EM framework, we also implement and compare to switch EM but with PPF embedded (named sPPF-EM). We expand on the differences in section 4.5.3.

Once models are learned with different learning methods in both simulations and experimental data, we compare the quality of learned parameters from different methods by using these learned parameters to perform state estimation with either PPF or switching PPF on held-out test data. Note that the model structure is identical regardless of what learning method is used. Thus, all differences in test set evaluations can be attributed to the learning methods. We compare the accuracy of the learned parameters for decoding latent states and performing one-step ahead prediction of the neural activity, which we refer to as neural self-prediction metric (section 4.6.2). For switching systems, we also decode regime states to evaluate decoded regime state accuracy. We refer to these metrics as decoding metrics and in simulation also normalize them by the decoding metrics from ground truth parameters: the normalized metrics range from 0 indicating chance to 1 indicating true performance. This gives us information both on how well the learned parameters can explain the data and on how similar they are to ground truth parameters. See section 4.6 for further details on simulation and evaluation methods.

### 2.2. PCF yields high accuracy parameter learning in stationary simulations

**Figure 3.**
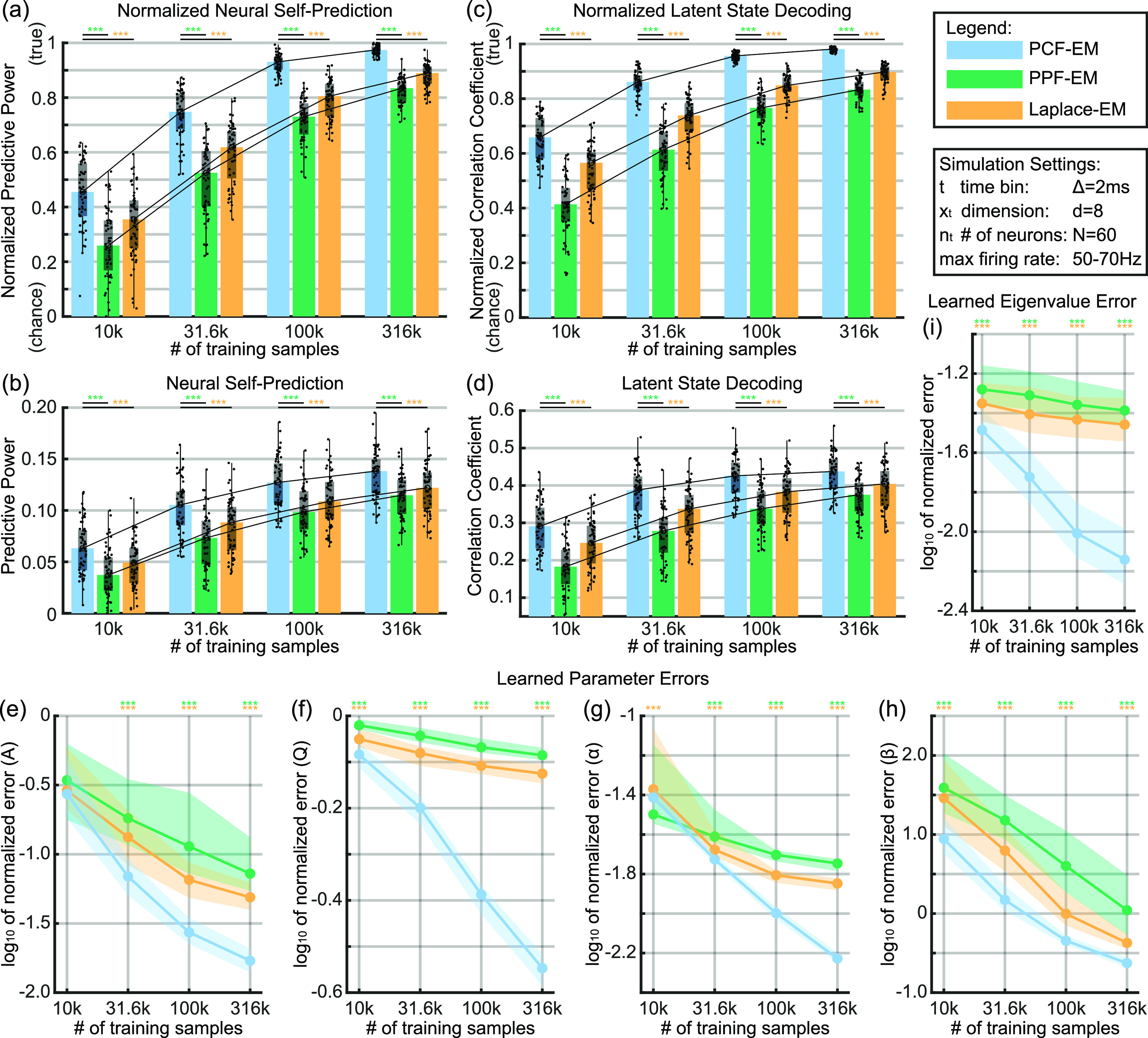
PCF leads to improved unsupervised learning capabilities in simulated stationary systems. (a) Neural self-prediction (predictive power, or ${\boldsymbol{PP}}$) on held-out test data using learned parameters normalized by that using ground truth parameters according to ${\boldsymbol{PP}}/{\boldsymbol{PP}_{\boldsymbol{true}}}$. Comparisons per training sample size are made between different learning methods: PCF-EM, PPF-EM, and Laplace-EM. Bars indicate the median metric, box edges indicate the 25th and 75th percentiles, and whiskers indicate minimum and maximum values excluding outliers that exceed 1.5 times the interquartile distance. Asterisks indicate statistical significance with ${}^{***}{\boldsymbol{p}} &lt; 0.0005$. Trend lines connect at the median. (b) Same as (a) but un-normalized. (c) Same as (a) but showing the latent state decoding (correlation coefficient, or ${\boldsymbol{CC}}$) normalized by the CC using ground truth parameters according to ${\boldsymbol{CC}}/{\boldsymbol{CC}_{\boldsymbol{true}}}$. Performance with learned parameters reaches 98% that with true parameters. (d) Same as (c) but un-normalized (e–h) normalized error of learned parameters with respect to true parameters over increasing training sample size. (i) Normalized error of learned eigenvalues of ${\mathbf{A}}$. For each panel and statistical test, ${\boldsymbol{n}} = 60$.

**Figure A1.**
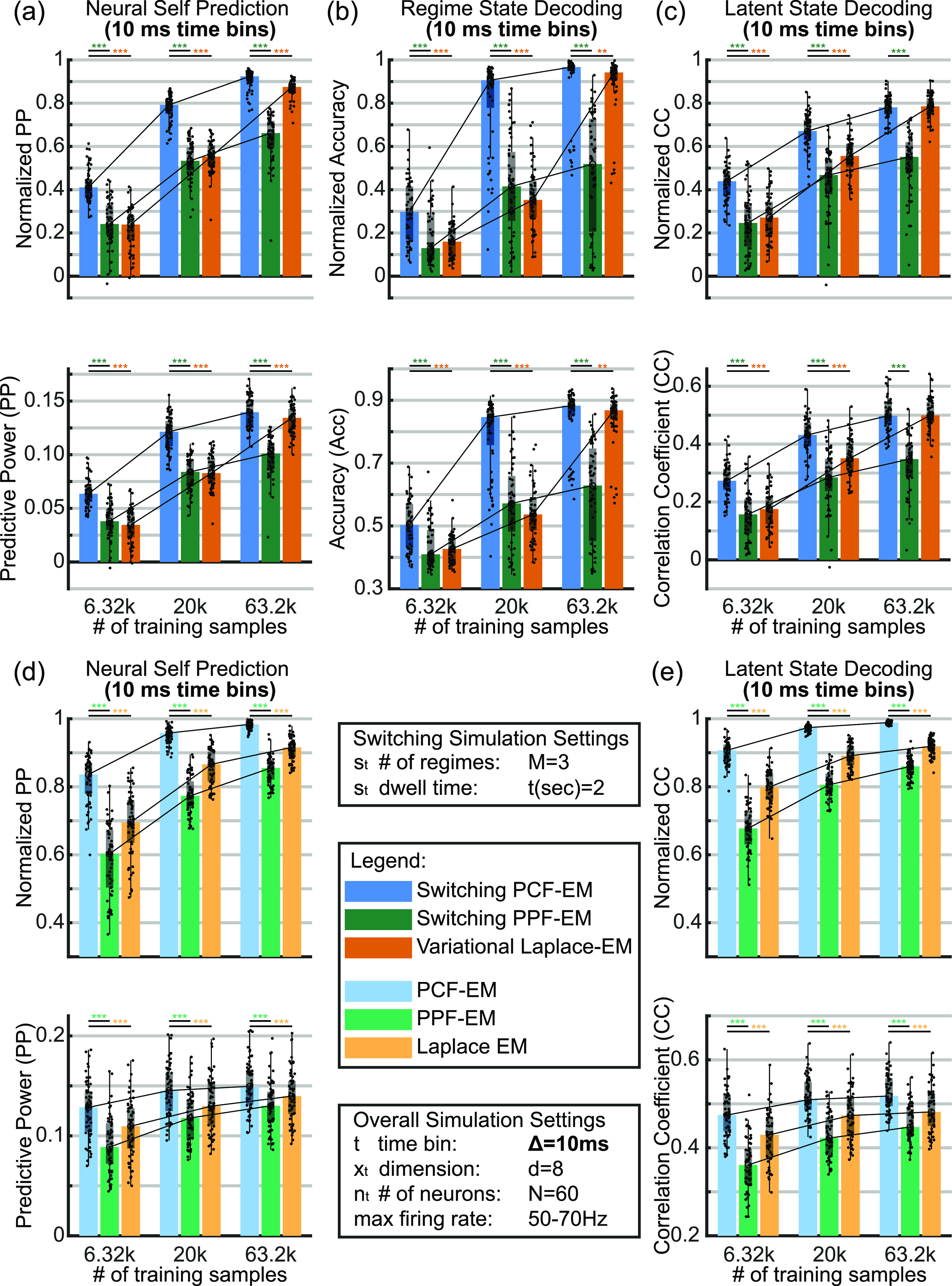
PCF successfully learns parameters for both stationary and switching systems even with larger time bins. Simulations use 10 ms time bins compared to the 2 ms time bins of figures 3 and 4. Bar, box, whisker, outlier, and asterisk conventions are the same as in figure 4. (a)–(c) Decoding metrics on held-out switching test data similar to figures 4(a)–(h) but with 10 ms time bins. Top panels show normalized metrics while bottom panels show un-normalized. As in figure 4, decoding metrics with learned parameters from PCF trend towards those with ground truth parameters, indicating successful learning. (d), (e) Decoding metrics on held-out stationary data similar to figures 3(a)–(d) but with 10 ms time bins. In addition to the 2 ms time bins, PCF-based learning also successfully learns parameters for the 10 ms time bins.

We first validate in simulations of stationary systems that PCF-EM can successfully learn accurate system parameters. We find that the latent state decoding and neural self-prediction metrics reaches 98.1% and 97.4% of that using ground-truth system parameters, respectively (figures 3(a) and (c)). Figures 3(a) and (c) further demonstrate that these decoding metrics converge toward the performance with true parameters as more training samples are provided, indicating learned parameters approaching ground truth values. While these results are for systems simulated with 2 ms time bins, we find the same trends with an increased time bin size of 10 ms as shown in figures A1(d) and (e). Also, the learned parameter error decreases with increasing training data size as shown in figures 3(e)–(i), again indicating successful parameter learning with PCF-EM (section 4.6.2). Finally, we find that PCF-EM significantly outperforms prior learning methods LEM and PPF-EM as seen throughout figure 3. In terms of learned parameter error, PCF-EM yields a 79.7% decrease in eigenvalue error compared to PPF-EM as shown in figure 3(i). In terms of decoding metrics, PCF-EM yields a 9.16% increase in latent state correlation coefficient (CC) over LEM in the largest training sample size case as shown in the rightmost cluster of figure 3(c). We emphasize that all learning methods use the same EM framework but differ in what inference method is used for the E-step as described in section 4.4; also, the evaluation of learned parameters uses the same pipeline with PPF as described in section 4.6.2. As such, all improvement can be attributed to the utilization of the new PCF for learning.

### 2.3. PCF yields high accuracy parameter learning in switching simulations

**Figure 4.**
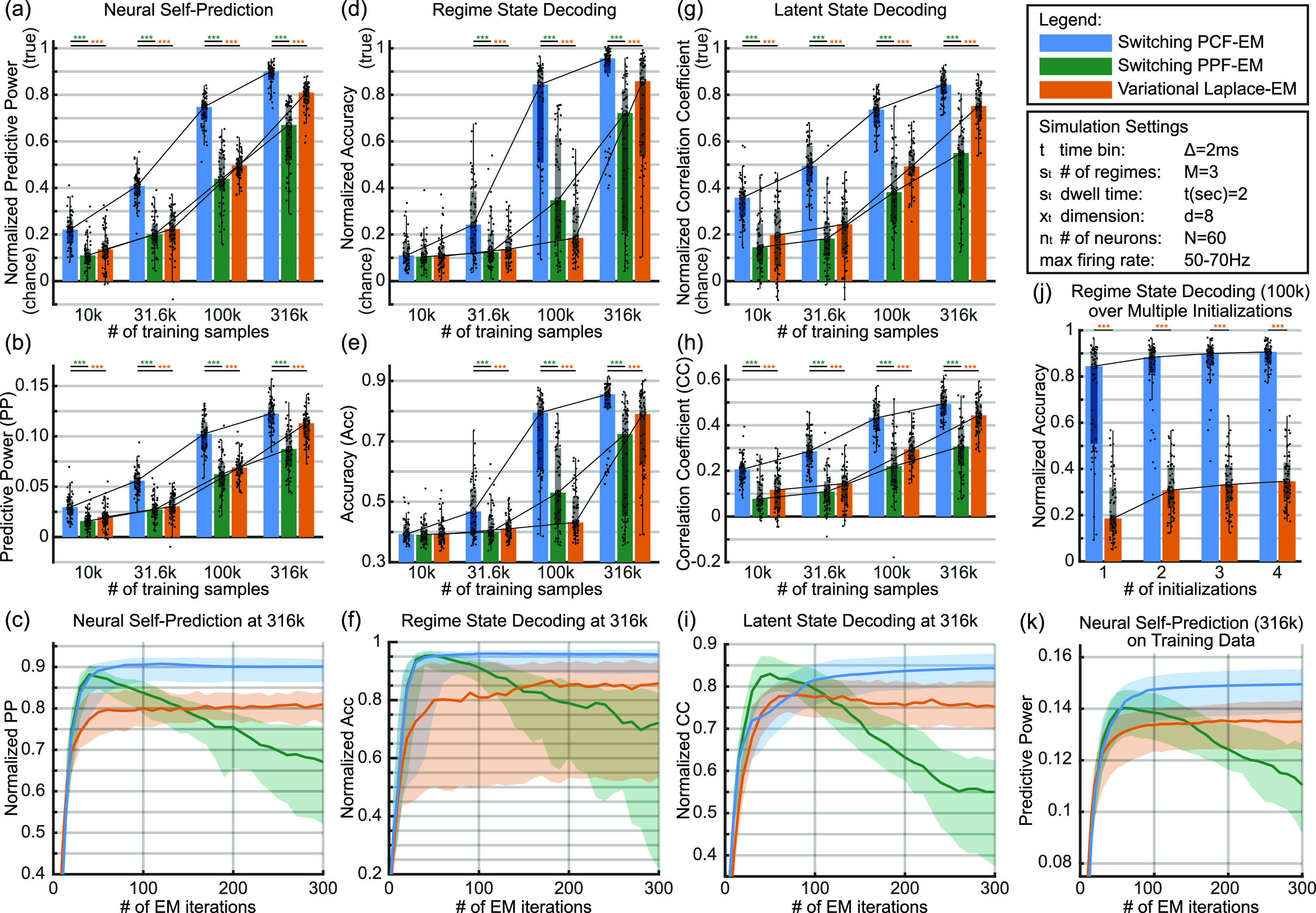
PCF leads to improvements in unsupervised learning in simulated switching dynamical systems with Poisson observations and 2 ms time bins. Bar, box, whisker, outlier, and asterisk conventions are the same as in figure 3. (a) Neural self-prediction on held-out test data using learned parameters normalized by that using ground truth parameters over an increasing number of training samples. Comparisons are made between different learning methods: sPCF-EM, sPPF-EM, and vLEM. (b) Same as (a) but un-normalized. (c) Normalized neural self-prediction over EM iterations at the largest training sample size of 316 k. (d) Regime state decoding on test data using learned parameters normalized by that using true parameters according to $\left( {\boldsymbol{Accuracy} - \frac{1}{\boldsymbol{M}}} \right)/\left( {{\boldsymbol{Accuracy}_{\boldsymbol{true}}} - \frac{1}{\boldsymbol{M}}} \right)$ where ${\boldsymbol{M}}$ is the number of regimes such that 0 is chance and 1 is true parameter performance. (e) Same as (d) but un-normalized. (f) Normalized accuracy over EM iterations. (g)–(i) Same as (a)–(c) but for latent state decoding with learned parameters. (j) Normalized regime state decoding accuracy using parameters trained with 100 k samples as a function of available initializations. After learning each initialization to completion (300 EM iterations), parameters associated with the initialization with the highest neural self-prediction in training set are chosen. (k) Neural self-prediction in training set at 316 k samples over EM iterations. While sPCF-EM and vLEM show consistent growth or convergence as expected of EM frameworks, sPPF-EM shows divergence showing the critical need for PCF to enable a switch EM framework. For each panel and statistical test, ${\boldsymbol{n}} = 60$.

We then find that PCF enables accurate parameter estimation for switching dynamical systems through a switch EM learning framework (sPCF-EM). Similar to section 2.2, as more training samples are provided in learning, the evaluation decoding metrics trend towards those obtained from filters using ground truth parameters as seen in figures 4(a),(d) and (g). Indeed, the neural self-prediction and regime state decoding metrics reach 90% of the performance using true parameters while latent state decoding reaches 84%, with more training samples expected to further increase these values. Similar trends are found even with increased bin sizes as shown in figures A1(a)–(c). Thus, the ability for the parameters learned by sPCF-EM to explain data and infer unobserved states approaches that of the ground truth parameters, indicating successful parameter learning.

We also find that PCF is critical in enabling a successful switch EM learning framework for Poisson observations by implementing and comparing to a switch EM that instead uses a PPF (sPPF-EM). Note that a successful switch EM even with a PPF has not been demonstrated before, and we implement this here. We find that using the PCF instead of PPF in the switch EM yields at least a 31% increase in all normalized decoding metrics—neural self-prediction, regime decoding, and latent state decoding—for the largest training sample size case. This improvement is attributed to PCF because both sPCF-EM and sPPF-EM use the same switch EM framework with the only difference being whether PPF or PCF is embedded in the switching filter as described in section 4.5.3. Further, the local Laplace approximation of PPF when embedded in a switching filter in sPPF-EM can even result in diverging performance as seen in figure 4(c). We verify that this is not due to overfitting as decoding metrics on even the training data diverges as seen in figure 4(k). Thus, replacing the Laplace approximation with the more accurate deterministic sampling methods of PCF in the switching filter [1] is key to enabling a successful switch EM framework for unsupervised learning.

**Figure 5.**
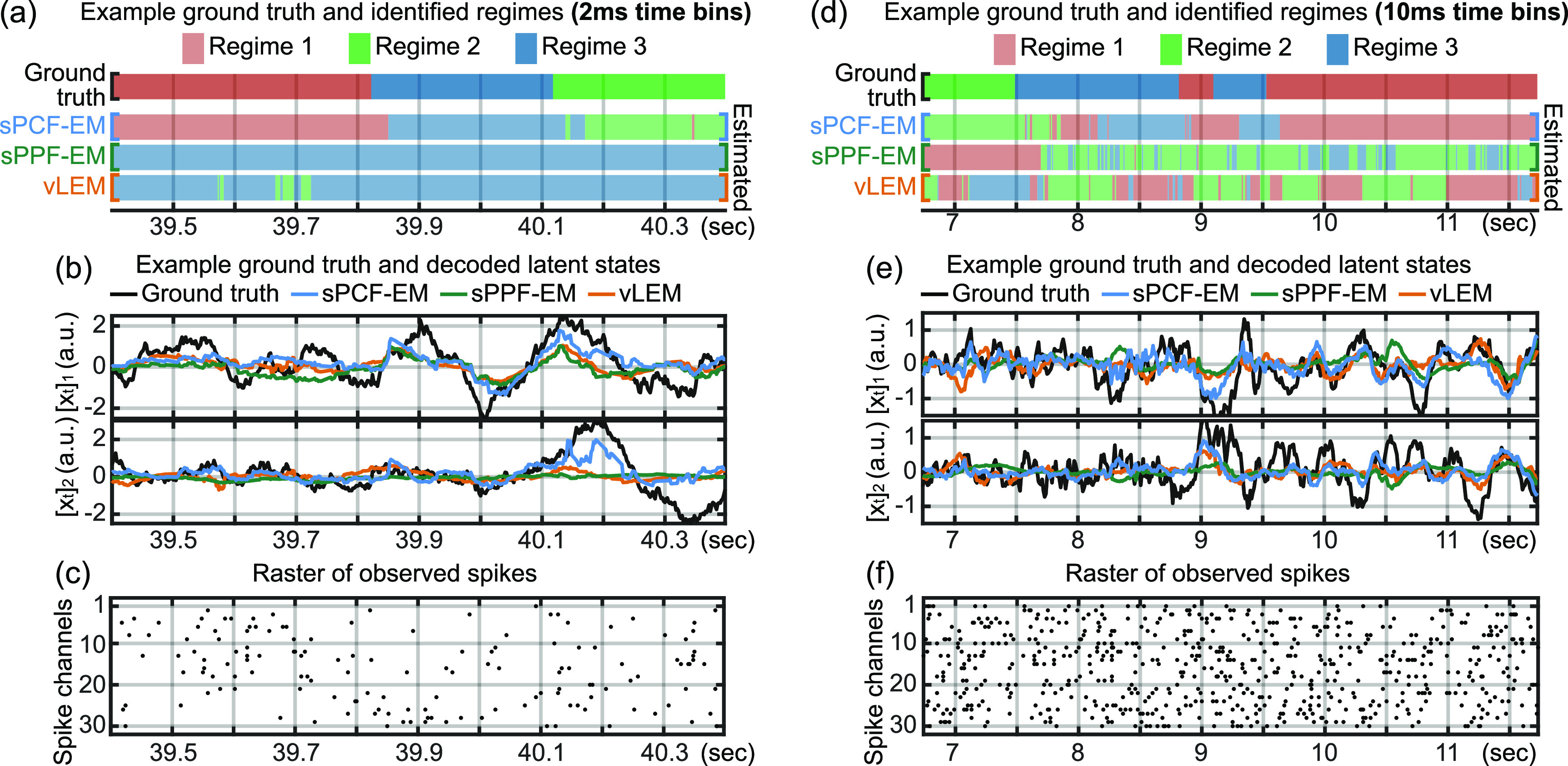
Visualizations of simulated data show successful decoding of latent and regime states with sPCF-EM. Spike data are binned in 2 ms and 10 ms time bins in the left and right columns, respectively; parameters are learned in both the 2 ms and 10 ms time bin cases using the same duration of data, amounting to 100 k samples and 20 k samples respectively. After parameters are learned with sPCF-EM, sPPF-EM, or vLEM in the training set, they are used in a switching PPF for test set decoding. (a)–(d) A 1 s segment from the test set of a simulated 3-regime switching system with 2 ms time bins, corresponding to 500 timesteps. (a) Ground truth and decoded regimes from the 3-regime system with each color indicating one regime. sPCF-EM’s data efficiency enables high accuracy decoding compared to sPPF-EM and vLEM. (b) Ground truth and decoded latent states (2 of the 8 latent dimensions are visualized). (c) Raster of observed spikes from 30 of the 60 simulated neurons. (d)–(f) Same visualization as (a)–(c) but for a 5 s segment from the test set of a simulated system with 10 ms time bins. As the time bins are longer, (d)–(f) also visualize 500 timesteps.

We also find that sPCF-EM outperforms the prior vLEM method [3] which embeds global Laplace in a variational EM framework [28, 29]; indeed, sPCF-EM can more than double the normalized regime decoding accuracy for certain data lengths compared with vLEM. As can be seen in figures 4(a) and (g), sPCF-EM learns parameters that yield significantly higher neural self-prediction and latent state decoding compared to vLEM across all training sample sizes with 2 ms bin sizes. Critically, we also find in figure 4(d) that sPCF-EM is more data sample efficient than vLEM, which is critical for neural datasets as training samples are often limited. Specifically, sPCF-EM is able to reach 80% of normalized regime decoding accuracy with only 100 k samples compared to 316 k samples in vLEM (see third vs. fourth clusters of figure 4(d)). Also, at 100 k samples, normalized regime decoding accuracy with vLEM is lower than 20% (figure 4(d), third cluster), suggesting that in some cases, regimes that could otherwise have been uncovered with sPCF-EM would not be found by vLEM. This data efficiency result also holds when increasing the bin size to 10 ms as shown in figure A1(b). Here, sPCF-EM reaches above 90% normalized accuracy compared to 35.3% for vLEM in the middle cluster of training samples. This result suggests that sPCF-EM maintains a data efficiency advantage over vLEM even with changes to bin size. Visualizations of these cases for both bin sizes are shown in figure 5. Overall, these results show that sPCF-EM is especially important for identifying regimes under limited data length constraints as can be the case when working with experimental brain data. These results collectively suggest that using PCF over global Laplace can improve learning, as was also seen in stationary systems. Beyond switch EM, an interesting future direction would be to explore how PCF can be incorporated into the variational EM frameworks to potentially enable higher accuracy and more data efficiency (see section 3).

We also find that the variability of performance can be further reduced by trying more initializations in sPCF-EM. Initializations can be important to help EM avoid being stuck in local maxima or saddle points [30, 35]. We thus explore increasing the number of initializations for this 100 k training sample size. We find that as we increase the number of initializations and choose the one with the best neural self-prediction within the training set, we can reduce the spread of the regime decoding accuracy such that even the 25th percentile of it exceeds 80% of the true parameter performance using 3 initializations as can be seen in figure 4(j). We repeat this procedure for vLEM with the same initializations that were used for sPCF-EM and find that while its regime decoding accuracy also improves with the number of initializations, the median performance saturates at 35% of the true parameter performance for vLEM.

### 2.4. PCF enables learning switching dynamical systems that better predict motor cortical data and better decode movement

**Figure 6.**
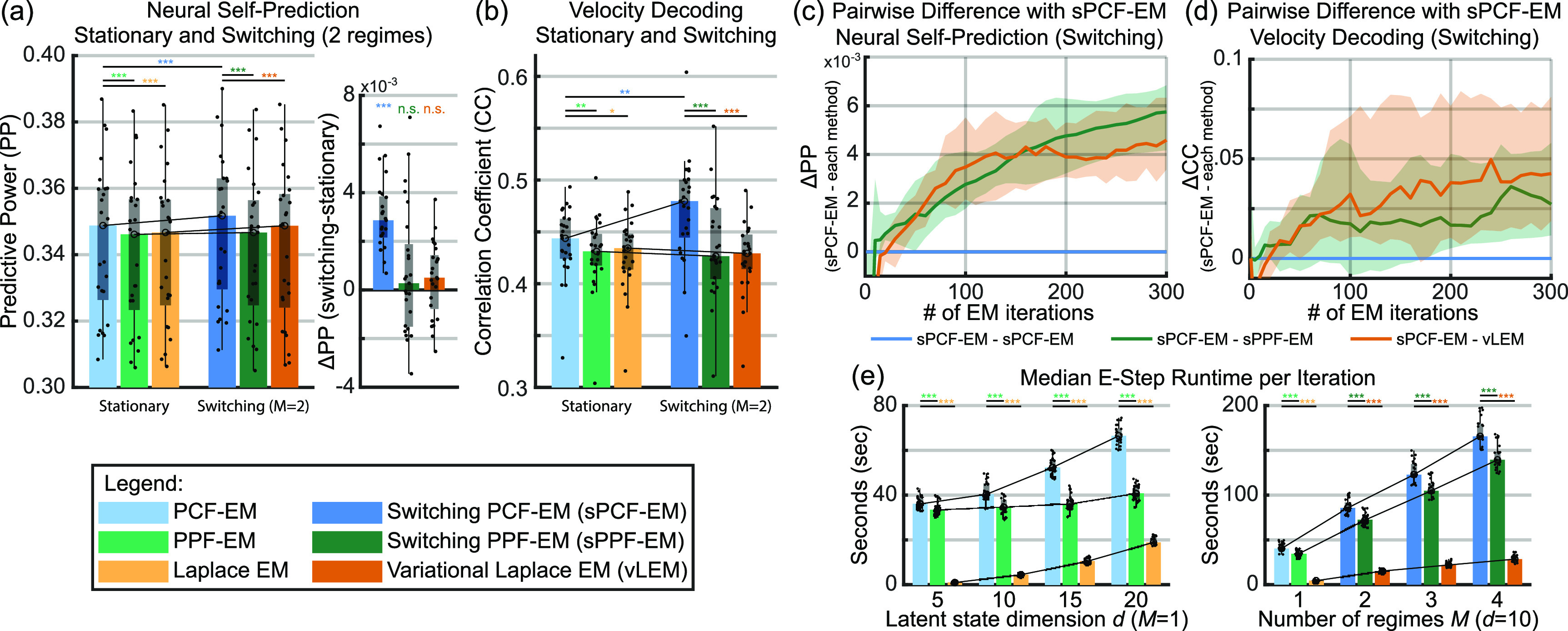
PCF improves quality of learned parameters in experimental data for both stationary and switching system models and demonstrates the increased explanatory power of switching systems over stationary systems. Plotting conventions like bar, box, whisker, and outliers are the same as in figure 3. (a) Left shows neural self-prediction on held-out test data from parameters learned with methods listed in the legend. The first cluster is for stationary methods while the second cluster is for switching methods with the number of regimes ${\boldsymbol{M} = 2}$. Note that all metrics within a cluster are obtained from the same filter. Right shows pairwise differences between stationary methods and their respective switching methods. Only switching systems that are learned with PCF show significant improvements in neural self-prediction over stationary systems. (b) Decoding of behavior (2D reach velocity) on held-out test data across methods. (c) Pairwise differences in neural self-prediction of test sets between sPCF-EM and either sPPF-EM or vLEM over iterations. Higher values indicate degree to which sPCF-EM outperforms sPPF-EM and vLEM. (d) Same as (c) but with pairwise differences in velocity decoding. (e) Median runtime of E-step over sweeping latent state dimension (left) and sweeping number of regimes (right). For statistical tests, ${\boldsymbol{n} = 25}$, ${}^{*}{\boldsymbol{p}} \unicode{x2A7D} 0.05$, ${}^{**}{\boldsymbol{p}} \unicode{x2A7D} 0.005$, ${}^{***}{\boldsymbol{p}} \unicode{x2A7D} 0.0005$.

We apply sPCF-EM to publicly available population spiking activity [41] recorded from the motor cortical areas of a monkey during a continuous 2D point-to-point reach task binned using 10 ms time bins (see figure 2 and section 4.7.1 for details on specific sessions and data sizes). First, we find that a switching dynamical system learned with sPCF-EM explains the data better than a stationary dynamical system (figure 6). To first fix the latent state dimension, we learn stationary systems for a range of latent dimensions. We find that the cross-validated median neural self-prediction reaches 99% of the peak value at a latent dimension of 10, which we fix for subsequent analyses. We then compare decoding metrics from learned stationary systems to learned switching systems with 2 regimes in a five-fold cross-validation scheme for each of the 5 experimental sessions (see section 4.7). As further increasing the number of regimes does not yield significant improvements to decoding metrics, we focus on the 2-regime case. We find that switching systems learned with sPCF-EM not only yield significantly higher neural self-prediction compared with stationary systems, but also yield a significant 8% increase in behavior decoding as seen in figures 6(a) and (b).

**Figure A2.**
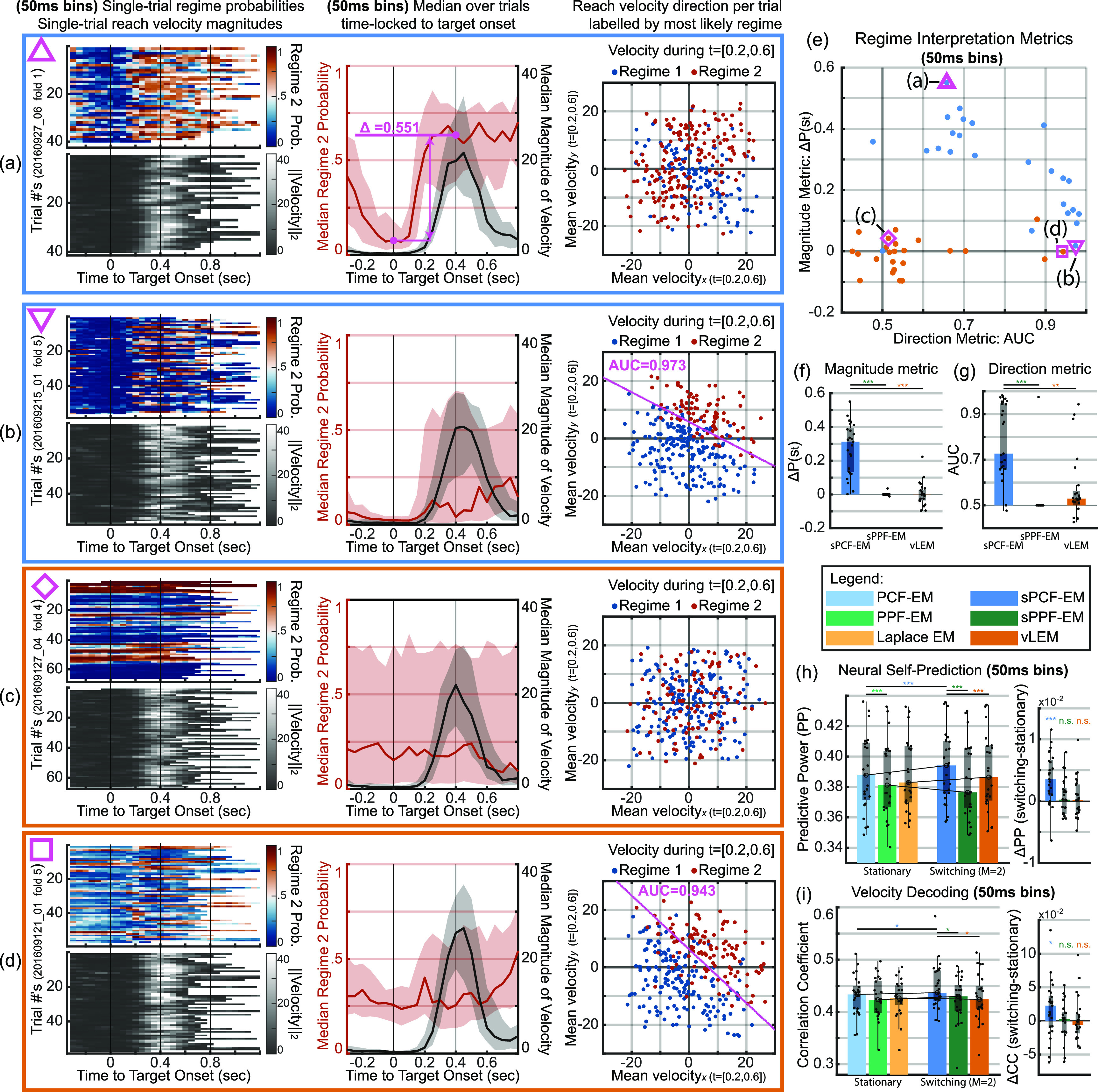
PCF can also uncover interpretable behavior-related regimes with larger 50 ms time bins. Bar, box, whisker, outlier, and asterisk conventions are the same as in figure 6. Analyses and plot conventions for (a)–(d) are consistent with (a)–(c) of figure 7 except that the analyses in figure 7 use smaller 10 ms time bins. (a) Regime interpretation of sPCF-EM for a representative fold displaying regimes corresponding with reach magnitude. Middle panel shows median regime probability (red) time-locked to target onset and rising sharply with reach magnitude, consistent with figure 7(a) but with a coarser timescale. (b) Regime interpretation of sPCF-EM for a representative fold displaying regimes corresponding with reach direction. (c) Regime interpretation of vLEM for a representative fold, showing regimes corresponding to neither reach magnitude nor direction. (d) One of the few folds of 25 folds analyzed with vLEM that show task-relevant regimes corresponding to reach direction. (e) Scatter of magnitude metrics against direction metrics across all analyzed folds for sPCF-EM (blue dots) and vLEM (orange dots). sPCF-EM more reliably uncovers regimes that largely indicate either switches in direction or switches in magnitude compared to vLEM whose metrics are largely clustered around chance-level for both metrics. (f) Magnitude metric across folds. (g) Direction metric across folds. (h) Neural self-prediction on held-out test data for stationary (left cluster) and switching (right cluster) models across folds. Right panel shows pairwise differences between stationary methods and their respective switching methods. (i) Same as (h) but with velocity decoding. For statistical tests, ${\boldsymbol{n}} = 25$, ${}^{*}{\boldsymbol{p}} \unicode{x2A7D} 0.05$, ${}^{**}{\boldsymbol{p}} \unicode{x2A7D} 0.005$, ${}^{***}{\boldsymbol{p}} \unicode{x2A7D} 0.0005$.

Second, similar to our simulation results, we also find that switching systems learned with sPCF-EM outperform switching systems learned with sPPF-EM and vLEM in terms of both neural self-prediction and behavior decoding (figures 6(a) and (b)). This result holds as well for models learned on the same data but with larger 50 ms time bins, with sPCF-EM significantly outperforming both sPPF-EM and vLEM (figures A2(h) and (i)). For example, in the 10 ms time bin case, switching systems learned with sPCF-EM yield a 12% increase in velocity decoding CC over those learned with vLEM as seen in the right half of figure 6(b). These improvements in explanatory power with PCF-based methods require higher computation time as seen in figure 6(e), giving rise to a speed-accuracy tradeoff. We can attribute this increase in computational cost to the evaluations of the likelihood mean and covariance at the deterministically sampled points which scale in number polynomially with latent state dimension (see section 4.1). However, PCF-based methods have the possibility for more efficient implementations through parallelization (see Discussion) which was not the focus in this work. In exchange for this trade-off, in both simulation and experimental data, unsupervised EM learning with PCF yields higher quality parameters in terms of neural self-prediction and behavior decoding compared to prior Laplace-based methods. Further, sPCF-EM reveals behavior-relevant neural regimes that can otherwise be missed by these prior Laplace-based methods as we show next.

### 2.5. PCF enables discovering interpretable task-relevant neural population regimes in motor cortical data

**Figure 7.**
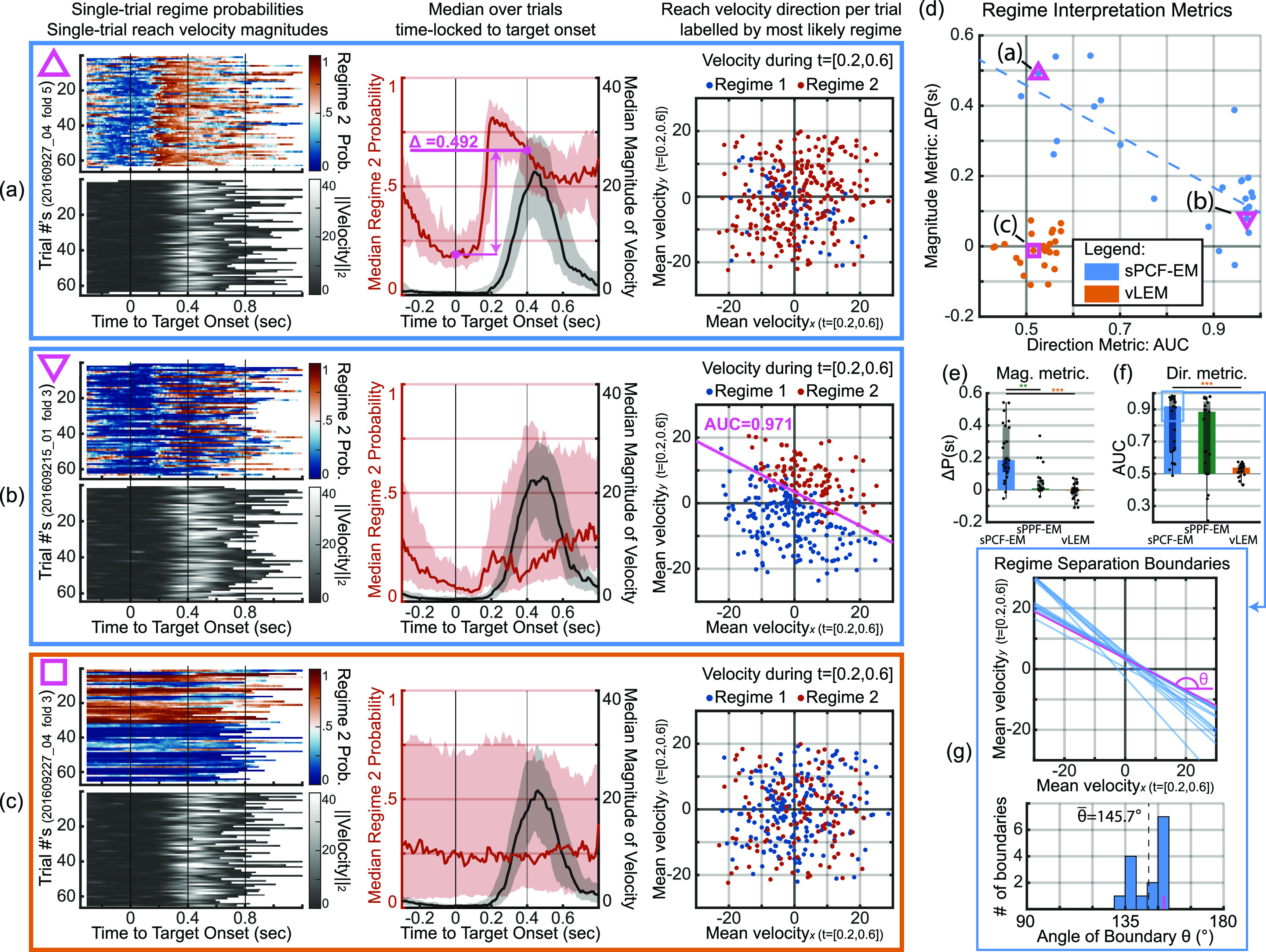
PCF enables uncovering interpretable behavior-related neural regimes, completely unsupervised with respect to behavior. Bar, box, whisker, outlier, and asterisk conventions are the same as in figure 6. (a) Regime interpretation of sPCF-EM for a single representative fold. Uncovered regimes correspond with reach magnitude. Left panel shows single-trial regime probabilities and reach velocity magnitudes from held-out test fold time locked to target onset with values indicated by their respective color bars. Note that the signals are continuous when not time locked. Middle panel overlays median regime probability (red) over median reach magnitude (black). For this fold, regime probability sharply rises on target onset and yields a difference in probability of 0.492 at 400 ms post onset vs. at onset. This metric will be called the magnitude metric as described in the text. Right panel shows a scatter of average ${\boldsymbol{x,y}}$ velocity during the period of expected movement per trial (200–600 ms post target onset) over the entire session. Points are labeled by most likely regime over the same period (regime with highest average decoded probability over 200–600 ms). Most points in this movement period are associated with regime 2 (red), showing regime 2’s relevance to movement. (b) Same analyses as in (a) from sPCF-EM but for a representative fold with regimes corresponding with reach direction. Right panel shows clear separation between labeled regimes with AUC of 0.971. This AUC will be called the direction metric as described in the text. Regimes separate to discriminate a top-right direction vs. a bottom-left direction, with the most discriminant linear boundary overlayed in magenta. (c) Same analyses as in (a, b) but for a representative fold using vLEM. Regimes describe neither reach magnitude nor reach direction, indicating the vLEM does not learn interpretable task-relevant regimes. (d) Scatter of magnitude metrics against direction metrics across all analyzed folds for sPCF-EM and vLEM. Representative folds of panels a and b are highlighted with magenta triangles and representative fold of panel c is shown with a magenta square. sPCF-EM reliably uncovers regimes that largely indicate either switches in direction or switches in magnitude. For vLEM, learned regimes describe neither reach magnitude nor direction across all analyzed folds. (e) Magnitude metric across folds. (f) Direction metric across folds. (g) Regime separation boundaries as in the right panel of (b) for all folds using sPCF-EM with high direction metric (AUC > 0.8). Bottom shows the histogram of the angles of these boundaries. Regimes that describe reach direction consistently yield a top-right and bottom-left separation boundary with a mean boundary angle of 145.7°. For statistical tests, ${\boldsymbol{n}} = 25$.

**Figure 8.**
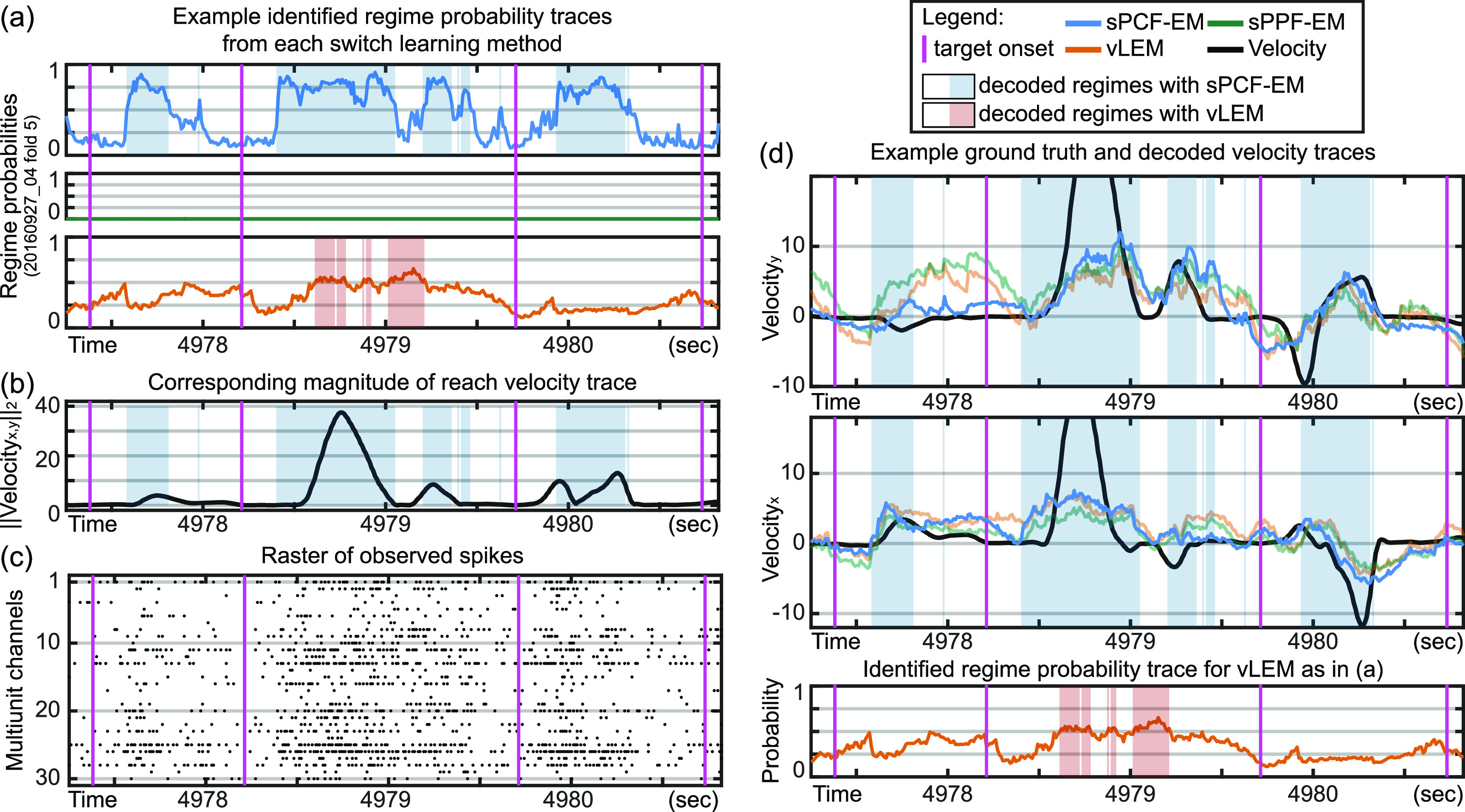
Visualization of a continuous segment of experimental data showing switches in decoded regimes learned by sPCF-EM, which correspond to changes in reach velocity magnitude. (a) Example decoded regime probabilities using parameters learned from each switching method: sPCF-EM, sPPF-EM, and vLEM. In these analyses, all methods have a total of two regimes. Magenta lines indicate target onset times. White patches indicate that regime 1 is decoded and colored patches indicate that regime 2 is decoded. For vLEM (orange), its two regimes describe the activity during this window roughly similarly, resulting in probabilities that do not confidently switch towards 0 or 1 for either regime. sPPF-EM (green) fails to identify a second regime for this fold and thus its decoded probability of regime 2 remains largely at 0. Velocity decoding would then be expected to perform similarly to a stationary system with the first regime’s parameters. (b) Magnitude of reach velocity over the same segment as in (a). Blue patches that correspond to decoded regime 2 from sPCF-EM largely correspond to increases in reach velocity magnitude as first explored in figure 7(a), showing that sPCF-EM can identify magnitude-related regimes. In comparison, orange patches that correspond to decoded regime 2 from vLEM are not magnitude-related. (c) Raster plot of multiunit activity over the same segment as (a). (d) Ground truth and decoded reach velocity traces for *x* and *y* reach velocity. Blue patches show the decoded regime 2 from sPCF-EM. Bottom panel reproduces the decoded regimes from vLEM for ease of visualization and is identical to the bottom panel of (a).

**Figure A3.**
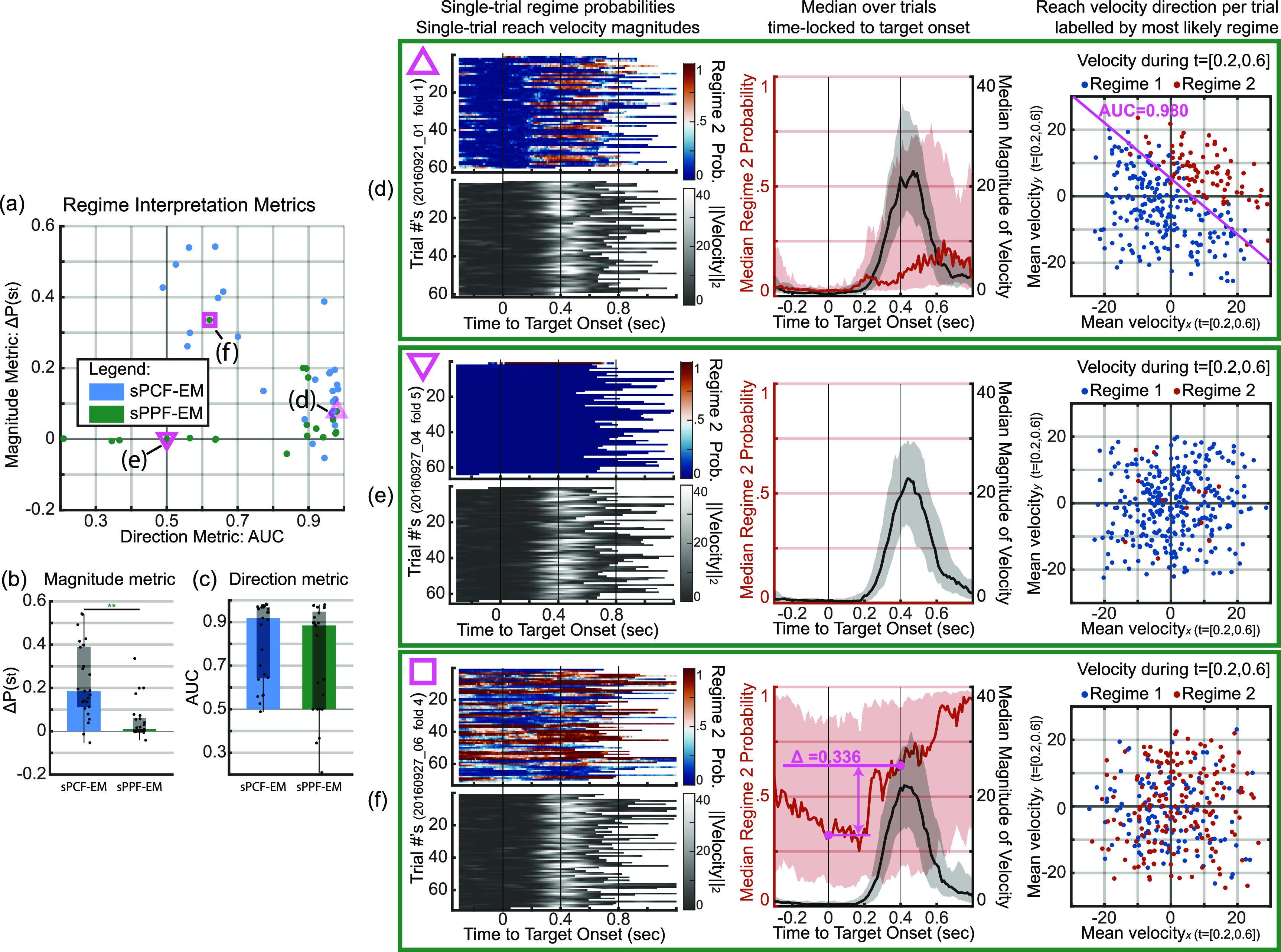
sPPF-EM can be unreliable in uncovering behavior-related regimes. Bar, box, whisker, outlier, and asterisk conventions are the same as in figure 6. Analyses and plot conventions for (d)–(f) are consistent with (a)–(c) of figure 7 with all three panels visualizing folds from sPPF-EM. (a) Scatter of magnitude metrics against direction metrics across all analyzed folds for sPCF-EM (blue dots) and sPPF-EM (green dots). Unlike sPCF-EM, sPPF-EM fails at identifying magnitude regimes while it can identify direction regimes similar to sPCF-EM. (b), (c) Magnitude and direction metrics across folds for sPCF-EM and sPPF-EM as reproduced from figures 7(e) and (f). (d) Regime interpretation of sPPF-EM for a representative fold displaying regimes corresponding with reach direction. (e) Regime interpretation of sPPF-EM for a representative fold displaying regimes that fail to capture switches in either direction or magnitude. Parameters learned with sPPF-EM can result in one regime being dominant such as regime 1 here. (f) There was a single outlier fold from sPPF-EM with a larger magnitude metric; however, even this outlier fold did not consistently indicate switches in magnitude across trials as can be seen by the much wider shaded area around the median regime probability in the middle panel here compared with that in figure 7(a) (shaded area represents 25th to 75th percentile of decoded regime probability across trials). For statistical tests, ${\boldsymbol{n}} = 25$, ${}^{**}{\boldsymbol{p}} \unicode{x2A7D} 0.005$.

We then find that despite being unsupervised with respect to behavior, sPCF-EM uncovers regimes in neural population activity that correspond to interpretable periods in the reaching behavior. As the task is continuous and self-paced, we first visualize single-trial decoded regime probabilities by time-locking the probabilities and reach velocity magnitudes to target-onset event labels per test fold (figures 7(a)–(c)). Through this, we find that several of the analyzed folds exhibit a regime pattern that corresponds to reach velocity magnitude. Empirically, one regime is dominant while the monkey is holding on the current target and until it reacts to the onset of the next target (hold regime). A second regime then has its probability sharply rise at the onset of this next target (movement regime). A representative fold is shown in figure 7(a) with additional visualizations of a continuous segment from that fold shown in figure 8. Since our method is unsupervised with respect to task or behavioral variables and just looks at neural data, we define interpretability of a decoded regime as the regime being task-relevant, i.e., interpretable in the context of the task or behavior. We thus interpret a given regime as being task-relevant when the switches in that regime correspond to changes in task-relevant behavioral variables, in this case reach magnitude. We use what we call a magnitude metric to aid in interpretation by quantifying how well a learned switching model can capture switches in reach velocity magnitude. We find this metric for each model by finding the cross-validated median decoded regime probability at target onset where movement is minimal and at 400 ms post target onset where movement is expected, and then taking the difference. For example, in figure 7(a) we find that the decoded probability is highly different between these two periods, indicating the ease with which the learned switching model could distinguish the two periods. In contrast, the regimes found with the prior vLEM did not distinguish movement vs. hold periods (figure 7(c)). Across all models learned with sPCF-EM vs. vLEM, we find that sPCF-EM is significantly more reliable in uncovering regimes with higher magnitude distinguishability as seen in figure 7(e). We also find that PCF is key to enabling the switch EM framework to uncover these magnitude regimes more reliably; this is because the magnitude metrics from sPCF-EM is significantly larger than those from sPPF-EM, which we also implemented in this work (figure 7(e)) (note sPPF-EM has not been shown in prior work). One of the representative folds of sPPF-EM that fails to identify a magnitude regime is shown in figure A3(e).

Interestingly, for folds where the uncovered regimes did not clearly correlate with reach velocity magnitude, sPCF-EM instead uncovered regimes that better correspond to reach direction. To show this, we decode the regimes in the entire session using the learned parameters per training set and focus on the 200 ms to 600 ms movement period post target onset where reaches are expected. This window is chosen because the median increase in velocity magnitude is centered on this window as exemplified by the gray traces in the central panels of figures 7(a)–(c). We then find the average $x$, $y$ velocity in each trial in that period. We also separately label each trial by the regime with the highest average decoded probability in that time period which we emphasize was found unsupervised with respect to behavior. We then provide the scatter plot for the average $x$, $y$ velocity and label each point by the decoded regime. As shown in the rightmost panel of figure 7(b), we find that the decoded regimes can be clearly differentiated by average $x,y$ velocity. We also find that sPPF-EM is capable of identifying similar regimes with a representative fold shown in figure A3(d), although we emphasize that sPPF-EM fails to identify magnitude regimes unlike sPCF-EM (figures 7(e) and A3(a), (b)). Here, reaches in the bottom-left direction were largely identified as one regime by sPCF-EM (bottom-left regime) compared to reaches in the top-right direction (top-right regime).

We use what we call a direction metric to quantify how well a learned switching model can capture switches in reach direction. This metric is based on the differentiability of the decoded regimes by average $x,y$ velocity. We find this metric by calculating the mean AUC from an LDA classifier that predicts the decoded regime from average $x,y$ velocity over five folds. Here, an AUC of 0.5 indicates chance while 1 indicates perfect linear differentiability. We find the median AUC over the 25 models to be 0.92 with 10 of the 25 reaching above 0.95 AUC as shown in figures 7(d) and (f). Further, for models with AUC’s above 0.8, we find that the regimes do not discriminate arbitrary directions but rather they consistently discriminate a to p-right and bottom-left pattern as shown in figure 7(g). This result may suggest a consistent regime pattern in neural population dynamics that across all sessions distinguishes reaches towards the body versus away from the body.

Further, we find that the ability for sPCF-EM to uncover regimes is not dependent on the size of the time bins used in our analyses. More specifically, we replicated the above regime interpretation analyses in figure A2 but on models learned on data binned in 50 ms windows, which are five times larger than those used for figure 7. As illustrated in figure A2, even with larger time bins, sPCF-EM is able to uncover task-relevant regimes while being unsupervised. Here, representative folds in figures A2(a) and (b) illustrate regimes that correspond to reach magnitude and reach direction, respectively. The single-trial decoded probabilities in the left panels of figures A2(a) and (b) are also visually similar to those in figures 7(a) and (b), but with a coarser timescale due to the increased time bin size. The results of the larger bin size illustrate how sPCF-EM can flexibly extend to other time bin sizes because it is derived for a Poisson observation modality that allows for multiple spikes per bin.

Finally, we find that models from the prior vLEM method are significantly less reliable in capturing switches in either direction or magnitude in these experimental datasets regardless of time bin size. As seen in figures 7(e) and (f) for the 10 ms time bins, both direction and magnitude metrics for vLEM across all analyzed folds are clustered at chance level with a representative fold shown in figure 7(c). With the larger 50 ms time bins, the direction and magnitude metrics from vLEM across all folds are still significantly lower than those from sPCF-EM and are still clustered around chance level (figures A2(f) and (g)) with a representative fold shown in figure A2(c). Indeed, even in this larger time bin case for vLEM, the vast majority of folds had their direction and magnitude metrics around chance (figures A2(c), (f) and (g)) with just a few folds showing regimes corresponding to reach direction (see one such fold in figure A2(d)). This result suggests that vLEM is less reliable than sPCF-EM in identifying task-relevant regimes in these datasets. This result can be due to the sample efficiency of sPCF-EM compared with vLEM, which we also saw in simulations (figures 4(d) and A1(b)). While vLEM can be successful in decoding regimes in simulations (section 2.3), it needed substantially more training samples to do as well as sPCF-EM as seen in figure 4(d). Further, even when changing the bin size for the same duration of data, sPCF-EM still outperforms vLEM as seen in the 2nd cluster of figure A1(b). Together, these results suggest that sPCF-EM can be successful but vLEM can have difficulty in regime identification for the duration of data per session in these experimental datasets (section 4.7.1), similar to what we found in simulation. However, we emphasize that with sufficient data, vLEM is still a powerful tool and also offers good computational efficiency. Future work could investigate an integrated framework which can collectively leverage the individual benefits of PCF-based learning and variational methods.

## 3. Discussion

With the goal to enable accurate unsupervised learning of switching or stationary dynamical systems from Poisson observations, we developed a novel deterministic sampling filter for Poisson observations called the PCF to be used for EM learning. PCF includes novel derivations to enable an MMSE estimation approach using Poisson observations. It uses numerical integration techniques using a spherical–radial cubature rule to generate deterministic samples for estimating the necessary MMSE terms. We demonstrated how PCF can enable EM frameworks for accurate unsupervised learning of stationary (PCF-EM) and switching (sPCF-EM) dynamical systems using only Poisson spiking activity. We found that PCF enables more accurate learning of both stationary and switching dynamical systems compared with prior methods, thus largely improving the neural self-prediction, behavior decoding, and regime decoding in both simulations and motor cortical data from the primate brain during reaching movements. Also, (s)PCF-EM is more data-efficient than prior learning methods, which is important for experimental datasets. Finally, sPCF-EM enabled discovering regimes in the motor cortical population activity that were interpretable and behavior-relevant despite being learned fully unsupervised with respect to behavior. These interpretable neural population regimes were not found by prior learning methods.

We first validated the methods using numerical simulations. In stationary systems, we showed that parameters are successfully learned by PCF-EM and approach ground truth model parameters. We also showed that PCF-EM significantly outperforms prior methods that use the Laplace approximation. For switching systems, parameters are again successfully learned and the gain from sPCF-EM over prior methods is even greater compared with the stationary case. To show that successful learning depends on the PCF, we also implemented an EM framework with the PPF and showed that it significantly underperforms the PCF-based EM. Moreover, sPCF-EM can outperform the prior vLEM method, and importantly can better identify regimes at lower training sample sizes, i.e., sPCF-EM is more data-efficient. Numerical simulations gave us additional tools to probe the developed methods as true latent states, regime states, and model parameters are known in simulations. Prior works have similarly used simulated data to develop new methods for biotechnologies and neural engineering [1, 3, 15, 20, 23, 46–60].

PCF’s ability to enable the unsupervised learning of switching systems lends itself to multiple potential applications. For brain–machine interfaces (BMIs) whose goals are to restore lost motor function [19, 57, 58, 61–64], learning with sPCF-EM can improve the real-time decoding performance compared to stationary methods as we found in our results. Additionally, sPCF-EM could also be a valuable tool in the study of internal/mental states and how they impact behavior. Prior work has shown that changes in mental states such as stress, engagement, and attention can lead to changes in how subjects perform tasks and that these mental changes can be described with regimes [8, 65, 66]. However, in many cases, accurate and frequent labels of mental states may not be available. Unsupervised methods like sPCF-EM are potential tools to learn different regimes that could correlate with different mental states using just neural activity and without mental state labels for supervised training. Further, in the context of mental disorders, the learned switching models could be paired with causal switching filters to detect switches into regimes of aberrant activity in real-time and enable personalized therapies such as with deep brain stimulation [59, 62, 67–69].

The unsupervised discovery of regimes enabled by PCF-based EM can also serve as a tool to facilitate basic neuroscience investigations into neural population dynamics. For example, we found that there exist regime switches in motor cortical dynamics that may correspond to hold vs. movement and to movements toward vs. away from the body. These regimes were found fully unsupervised with respect to behavior yet were behavior-relevant and interpretable. Also, these regimes were more reliably found by PCF-based EM and were largely not found by the prior vLEM method, likely due to PCF-based EM’s data efficiency. Thus PCF-based EM provides a tool for basic neuroscience investigations.

The improvements to switch learning that PCF enables was driven by a transition from the Laplace-based approximations to deterministic sampling-based approximations. This was evident from substantial improvements over the same EM framework that instead of PCF used the widely used PPF which utilizes the Laplace approximation for Poisson observations. While global Laplace methods could outperform the local approach of PPF, PCF-based methods were still largely more accurate in learning. We hypothesize that this improvement was due to the general property of Laplace approximation not being able to always accurately capture first and second moments due to focusing on a single point, whereas deterministic sampling methods use multiple points to approximate second moment terms. However, a topic for future work would be to theoretically characterize the degree of inaccuracy in second moment terms and how much this impacts learning.

As discussed in sections 2.1 and 4.2, deterministic sampling-based non-linear filters like the UKF and CKF are widely used [30, 37, 39, 45, 70–72] but cannot be directly applied to Poisson observations, thus requiring a novel filter to be developed from a fundamental MMSE approach to properly incorporate Poisson observations which are nonconjugate with the Gaussian latent state. While we performed the derivation for Poisson observations, the derivation is general with the only requirement being that the likelihood mean and covariance of the observation modality given the latent state is known and can be evaluated. Future work could then explore extending this derivation on other observation modalities that do not fit traditional non-linear filters, such as the Bernoulli observations, and develop deterministic sampling-based methods for these modalities. For the Bernoulli modality, recent work has also explored using the Polya–Gamma augmentation to enable conditionally conjugate inference [47, 73]. Thus, future work on deterministic sampling-based methods for Bernoulli observations can compare its learning performance with that from methods that use the Polya–Gamma augmentation.

Our results in both simulated and experimental data show how PCF-based learning methods can yield high accuracy parameter estimates. However, there are additional future directions of extensions apart from the above two to advance these methods. First, a known general problem with EM-based learning methods is that they can converge to local instead of global maxima [30, 35, 74]. For learning methods for switching systems, this can manifest as regimes not being properly identified especially with insufficient data as seen for vLEM in our experimental results and for all methods in our simulation results with the shortest training sample size. While we showed and used a simple process of randomly initializing multiple times to improve consistency, future work could investigate more principled approaches to initialization, including but not limited to deterministic annealing [30, 35, 75]. Second, while we found that the PCF-based methods were more accurate than prior methods, there was a trade-off in terms of computational efficiency with the prior vLEM method performing significantly faster. Nevertheless, our use of a spherical–radial cubature rule in PCF instead of Gauss–Hermite quadrature (GHQ) rule does enable polynomial scaling instead of exponential scaling with latent state dimension as described in section 4.1. Further, at lower state dimensions, PCF’s runtime was comparable to that of PPF. As the PCF method involves independent evaluations of the same few functions over many deterministically sampled points before taking weighted sums, implementations that can more efficiently parallelize these evaluations could see meaningful reductions in runtime in the future. Third, while PCF was embedded in a switch EM framework here, future work could also explore using PCF in a variational learning framework, which may have potential benefits such as improved computational efficiency. Fourth, the model assumed for this work uses linear dynamics which offers benefits like interpretability, computational efficiency in both learning and inference, data efficiency, and extensions to real-time applications [2, 14, 19, 20, 61, 62, 67–69, 76–80]. Extending to switching linear dynamics, as we do here, can increase explanatory power and approximate nonlinearities while maintaining the above benefits of linear dynamical systems given that dynamics per regime are kept linear. Future work can explore extending the model and the associated learning methods to those with non-linear dynamics per regime to further increase explanatory power, though doing so may lead to a tradeoff in terms of interpretability or computation and data efficiency.

Finally, our results show how replacing the Laplace approximation in a causal filter with deterministic sampling can lead to improvements in learning, especially for switching systems. However, causal filters using the Laplace approximation are still valuable tools. First, we used PCF just for learning. Once model parameters are learned, we can use the faster filters based on Laplace approximation for decoding purposes with the learned parameters. For stationary systems, PPF that uses the Laplace approximation is known to be quite efficient and even implemented in real-time BMIs for millisecond-by-millisecond control and feedback [13, 48, 80–84]. For switching systems, recent work developed a switching filter that uses Laplace approximation [1] and showed that, once parameters are known, this filter can successfully perform causal decoding. Indeed, all decoding metrics in this work, once parameters are learned, use filters with Laplace approximation [1, 13, 16]. Thus, Laplace approximation-based filters serve as valuable companion methods to the developed PCF-based learning methods of this work, especially in the context of real-time decoding applications once parameters are learned with PCF-based learning.

Taken together, we developed PCF-based EM frameworks that achieve accurate unsupervised learning of stationary and switching dynamical system models from Poisson observations with both basic science and neurotechnology applications.

## 4. Methods

In this section, we introduce the tool of numerical integration based on cubature rules and provide details on how we use it in our approach to deriving the novel PCF. We then show how PCF can be embedded in unsupervised learning frameworks for both stationary and switching dynamical systems with figure 1 serving as a visual summary. We finally detail the validation methods including performance metrics, simulated data setup, and applications of our methods on experimental data.

### 4.1. Background: numerical integration based on spherical–radial cubature rules

We first briefly introduce the tool of numerical integration which we use to approximate important terms when deriving the PCF (see references in [37–39] for details). Numerical integration tools approximate solutions to complex integrals with weighted sums over a sparse set of points. One use case of this is to approximate expected values of functions of Gaussians since expected values of Gaussians are integrals over the Gaussian probability distribution. For PCF, we use the 5th-degree spherical–radial cubature rule of [38] to deterministically generate weights and points. As provided in [38], for a Gaussian random variable ${\mathbf{X}} \in {\mathbb{R}^{\boldsymbol{d}}}$ with mean and covariance ${\boldsymbol{\unicode{x03BC}}}$ and ${\boldsymbol{\Lambda }}$, the expected value of $g\left( {\mathbf{X}} \right)$ where $g\left( \cdot \right)$ is an arbitrary function can be approximated as:

\begin{align*}\begin{array}{*{20}{l}} {{E_{\mathbf{X}}}\left[ {g\left( {\mathbf{X}} \right)} \right] \approx \mathop \sum \limits_{i = 1}^{2{d^2} + 1} {w_i}g\left( {{\boldsymbol{\unicode{x03BC}}} + \sqrt {\boldsymbol{\Lambda }} {{\boldsymbol{\xi }}_i}} \right)} \\[3pt] {{w_1} = \frac{2}{{d + 2}}} \\[3pt] {{w_{2:2d + 1}} = \frac{{4 - d}}{{2{{\left( {d + 2} \right)}^2}}}} \\[3pt] {{w_{2d + 2:2{d^2} + 1}} = \frac{1}{{{{\left( {d + 2} \right)}^2}}}} \\[3pt] {{{\boldsymbol{\xi }}_1} = \boldsymbol{0}} \\[3pt] {{{\boldsymbol{\xi }}_{2:2d + 1}} = \left\{ { \pm \sqrt {d + 2} \cdot {{\mathbf{e}}_j}:j = 1,2, \ldots ,d{\text{ }}} \right\}} \\[3pt] {{{\boldsymbol{\xi }}_{2d + 2:2{d^2} + 1}}}\\[3pt] { \quad = \left\{ { \pm \sqrt {d + 2} \cdot \frac{1}{{\sqrt 2 }}\left( {{{\mathbf{e}}_k} \pm {{\mathbf{e}}_l}} \right):k &lt; l,k,l = 1,2, \ldots ,d{\text{ }}} \right\}\!.} \end{array}\end{align*}

where ${w_i}$ and ${{\boldsymbol{\xi }}_i} \in {\mathbb{R}^d}$ are the weights and points from the 5th-degree cubature rule. Here, $\sqrt {\boldsymbol{\Lambda }} $ can be found from the Cholesky decomposition of ${\boldsymbol{\Lambda }}$ and ${{\mathbf{e}}_j}$ are standard unit vectors. Further, these weights and points can be separately generated offline. See appendix section A.1. for an example point set for dimension $d = 3$. There exists alternative options for the weights and points such as the GHQ rule [39]. However, our choice of the cubature rule is important as the number of points given by GHQ scales exponentially with $d$, thus making it prohibitively expensive for high latent state dimensions. The 5th degree spherical cubature rule in comparison has polynomial scaling with $2{d^2} + 1$ points while offering comparable accuracy. We expand on this in appendix section A.1.

### 4.2. The model

We now detail the stationary form of the linear dynamical system model with Poisson observations which the filter is derived for with extensions to switching systems detailed in section 4.5. We start by modeling the latent brain state ${{\mathbf{x}}_t} \in {\mathbb{R}^d}$ which forms the latent representation of a behavior being performed as a random walk driven by zero mean Gaussian noise ${{\mathbf{w}}_t}$ with covariance ${\mathbf{Q}}$:

\begin{align*}{{\mathbf{x}}_t} = {\mathbf{A}}{{\mathbf{x}}_{t - 1}} + {{\mathbf{w}}_t}.\end{align*}

Here, the dynamics matrix ${\mathbf{A}}$ dictates how the latent state linearly evolves in time. The initial state ${{\mathbf{x}}_0}$ is assumed to be Gaussian with mean ${{\boldsymbol{\unicode{x03BC}}}_0}$ and covariance ${{\boldsymbol{\Lambda }}_0}$. The choice of using linear dynamics is motivated by the benefits they can provide in terms of interpretability, computational efficiency in both learning and inference, data efficiency, and ability to extend to real-time applications such as BMIs [2, 14, 19, 20, 61, 62, 67–69, 76–80]. Indeed, prior works in real-time neural engineering applications have shown that models with linear dynamics can enable accurate decoding and modeling of neural data [20, 67, 69, 78–80]. Moreover, our extension to switching linear systems can still maintain similar benefits due to linear dynamics per regime, while further increasing explanatory power (as seen in figure 6(b)) by approximating complex non-linear dynamics through a switching mechanism (see section 4.5). We refer to the parameters associated with the latent state dynamics in (7) as the dynamics equation parameters.

We use a Poisson observation modality to model recorded spiking activity. We assume for neuron $i$ that the latent state ${{\mathbf{x}}_t}$ is encoded in the instantaneous firing rate ${\lambda _i}$ and that the number of spikes within a time bin ${{\Delta }}$is Poisson distributed with rate ${\lambda _i}\left( {{{\mathbf{x}}_t}} \right){{\Delta }}$. With small-valued ${{\Delta }}$, this is equivalent to a point process observation modality [1, 13, 15–18, 22, 23, 46]. The conditional likelihood distribution of the spiking activity from $C$ neurons ${{\mathbf{n}}_t} = {\left[ {n_t^1, \ldots ,{\text{ }}n_t^C} \right]^T}$ is then:

\begin{align*}P\left( {{{\mathbf{n}}_t}|{{\mathbf{x}}_t}} \right) = \mathop \prod \limits_{i = 1}^C \frac{{{{\left( {{\lambda _i}\left( {{{\mathbf{x}}_t}} \right){{\Delta }}} \right)}^{n_t^i}}\exp \left( { - {\lambda _i}\left( {{{\mathbf{x}}_t}} \right){{\Delta }}} \right)}}{{n_t^i!}}{\text{ }}.\end{align*}

Here, we use a log-link function for the firing rate:

\begin{align*}{\lambda _i}\left( {{{\mathbf{x}}_t}} \right){{\Delta }} = \exp \left( {{\alpha _i} + {\boldsymbol{\unicode{x03B2}}}_i^T{{\mathbf{x}}_t}} \right).\end{align*}

We assume the spiking activity of separate neurons are conditionally independent of each other conditioned on the latent state ${{\mathbf{x}}_t}$ as consistent with prior works [13, 16–18]. As such, the likelihood mean and covariance of ${{\mathbf{n}}_t}$ is as follows:

\begin{align*}\begin{aligned} E\left[ {{{\mathbf{n}}_t}{\text{|}}{{\mathbf{x}}_t}} \right] &amp; = {\left[ {{\lambda _1}\left( {{{\mathbf{x}}_t}} \right){{\Delta }}, \ldots ,{\lambda _C}\left( {{{\mathbf{x}}_t}} \right){{\Delta }}} \right]^T} \hfill \\ V\left[ {{{\mathbf{n}}_t}{\text{|}}{{\mathbf{x}}_t}} \right] &amp; = {\text{diag}}\left( {{\lambda _1}\left( {{{\mathbf{x}}_t}} \right){{\Delta }}, \ldots ,{\lambda _C}\left( {{{\mathbf{x}}_t}} \right){{\Delta }}} \right) \hfill \\ \end{aligned} .\end{align*}

We refer to the parameters related to observation encoding in (8) and (9) as the observation equation parameters. Together, (7)–(9) constitute the model which is also referred to as a Poisson linear dynamical system model [3, 12, 14, 22, 23, 81].

As discussed in section 2.1, we cannot use existing deterministic sampling filters as the observation equation they are formulated for generally takes the form of ${{\mathbf{n}}_t} = h\left( {{{\mathbf{x}}_t}} \right) + {{\mathbf{v}}_t}$ where $h\left( \cdot \right)$ non-linearly but deterministically transforms the latent state and ${{\mathbf{v}}_t}$ is some additive noise. For the Poisson model though, the latent state is encoded in the firing rate (9) which dictates the probability of observing spikes in (8) rather than being a non-linear transformation into spikes. Therefore, we need to derive a new filter starting from fundamental estimation principles.

### 4.3. Derivation of the novel PCF

We now provide the derivation for the PCF. PCF’s goal in causal inference, also known as filtering, is to find an estimate ${\hat {\mathbf{x}}_t}$ of an unobserved state ${{\mathbf{x}}_{\mathbf{t}}}$ given Poisson observations up to the present time ${{\mathbf{n}}_{1:t}}$ in a recursive fashion. We denote the estimation of latent state ${{\mathbf{x}}_t}$ given observations ${{\mathbf{n}}_{1:\tau }}$ as ${\hat {\mathbf{x}}_{t|\tau }}$ with estimated state covariance similarly denoted as ${\hat {\boldsymbol{\Lambda }}_{t|\tau }}$. We assume at time $t - 1$ that the mean and covariance ${\hat {\mathbf{x}}_{t - 1|t - 1}}$ and ${\hat {\boldsymbol{\Lambda }}_{t - 1|t - 1}}$ of the posterior density $f\left( {{{\mathbf{x}}_{t - 1}}{\text{|}}{{\mathbf{n}}_{1:t - 1}}} \right)$ are known. The goal of a recursive filter is then to incorporate the next observation ${{\mathbf{n}}_t}$ and find the mean and covariance ${\hat {\mathbf{x}}_{t|t}}$ and ${\hat {\boldsymbol{\Lambda }}_{t|t}}$ of the posterior density at the next time step $f({{\mathbf{x}}_t}|{{\mathbf{n}}_{1:t}})$ to complete the recursion. As an intermediate step, we first find the mean and covariance ${\hat {\mathbf{x}}_{t|t - 1}}$ and ${\hat {\boldsymbol{\Lambda }}_{t|t - 1}}$ of the prediction density $f({{\mathbf{x}}_t}|{{\mathbf{n}}_{1:t - 1}})$ as follows because the model in (7) uses linear dynamics:

\begin{align*}\begin{array}{*{20}{l}} {{{\hat {\mathbf{x}}}_{t|t - 1}} = {\mathbf{A}}{{\hat {\mathbf{x}}}_{t - 1|t - 1}}} \\ {{{\hat {\boldsymbol{\Lambda }}}_{t|t - 1}} = {\text{ }}{\mathbf{A}}{{\hat {\boldsymbol{\Lambda }}}_{t - 1|t - 1}}{{\mathbf{A}}^T} + {\mathbf{Q}}{\text{ }}} \end{array}.\end{align*}

We note that we approximate $f\left( {{{\mathbf{x}}_{t - 1}}{\text{|}}{{\mathbf{n}}_{1:t - 1}}} \right)$ as Gaussian from the previous recursion and thus $f({{\mathbf{x}}_t}|{{\mathbf{n}}_{1:t - 1}})$ is also approximately Gaussian because the model uses linear dynamics.

The challenge in deriving the PCF then lies in the following measurement update step where we incorporate the new Poisson observation ${{\mathbf{n}}_t}$ and find the updated state estimate ${\hat {\mathbf{x}}_{t|t}}$ with covariance ${\hat {\boldsymbol{\Lambda }}_{t|t}}.$ If ${{\mathbf{n}}_t}$ was Gaussian and thus conjugate with the Gaussian prediction density, we could analytically find ${\hat {\mathbf{x}}_{t|t}}$ and ${\hat {\boldsymbol{\Lambda }}_{t|t}}$ using the Kalman update. However, because ${{\mathbf{n}}_t}$ is Poisson and nonconjugate with Gaussians, approximations are necessary. Therefore, we take a MMSE approach that is agnostic to the observation modality to find the estimate of the latent state ${\hat {\mathbf{x}}_{t|t}}$ that minimizes the expected mean-squared error with ${{\mathbf{x}}_t}$. In this approach, we constrain the estimator to be of the form ${\hat {\mathbf{x}}_{t|t}} = {\mathbf{M}}{{\mathbf{n}}_t} + {\mathbf{b}}$. Within this class of estimators, a known set of equations minimize the mean-squared error given by:

\begin{align*}\begin{aligned} {\hat {\mathbf{x}}_{t|t}} &amp; = {\hat {\mathbf{x}}_{t|t - 1}} + {{\boldsymbol{\Lambda }}_{{\mathbf{xn}}}}{\boldsymbol{\Lambda }}_{{\mathbf{nn}}}^{ - 1}\left( {{{\mathbf{n}}_t} - {{\hat {\mathbf{n}}}_{{\mathbf{t}}|{\mathbf{t}} - 1}}} \right) \hfill \\ {\hat {\boldsymbol{\Lambda }}_{t|t}} &amp; = {\hat {\boldsymbol{\Lambda }}_{t|t - 1}} - {{\boldsymbol{\Lambda }}_{{\mathbf{xn}}}}{\boldsymbol{\Lambda }}_{{\mathbf{nn}}}^{ - 1}{\boldsymbol{\Lambda }}_{{\mathbf{xn}}}^T \hfill \\ \end{aligned} \end{align*}

where the individual terms are as follows:

\begin{align*}\begin{aligned} {\hat {\mathbf{n}}_{t|t - 1}} &amp; = {E_{\mathbf{n}}}\left[ {{{\mathbf{n}}_t}{\text{|}}{{\mathbf{n}}_{1:t - 1}}} \right] \hfill \\ {{\boldsymbol{\Lambda }}_{{\mathbf{nn}}}} &amp; = {V_{\mathbf{n}}}\left[ {{{\mathbf{n}}_t}{\text{|}}{{\mathbf{n}}_{1:t - 1}}} \right] \hfill \\ {{\boldsymbol{\Lambda }}_{{\mathbf{xn}}}} &amp; = {E_{{\mathbf{x}},{\mathbf{n}}}}\left[ {\left( {{{\mathbf{x}}_t} - {{\hat {\mathbf{x}}}_{t|t - 1}}} \right){{\left( {{{\mathbf{n}}_t} - {{\hat {\mathbf{n}}}_{t|t - 1}}} \right)}^T}\left| {{{\mathbf{n}}_{1:t - 1}}} \right.} \right] \hfill \\ \end{aligned} .\end{align*}

For an estimator of the above form, these equations are known to minimize the mean squared error of the estimated state regardless of the observation modality distribution [2, 30, 39, 70, 85]. Thus, now the challenge of incorporating a nonconjugate observation modality becomes a challenge of how to find the terms in (13) for the Poisson observations. We emphasize with the subscripts ${\mathbf{n}}$ and ${\mathbf{x}},{\mathbf{n}}$ in ${E_{\mathbf{n}}}$ and ${E_{{\mathbf{x}},{\mathbf{n}}}}$ that these expectations are taken with respect to ${{\mathbf{n}}_t}|{{\mathbf{n}}_{1:t - 1}}$ and the joint density of ${{\mathbf{x}}_t},{{\mathbf{n}}_t}|{{\mathbf{n}}_{1:t - 1}}$, which are generally difficult to analytically calculate for nonconjugate observations. Importantly, however, from the prediction step, the density of ${{\mathbf{x}}_t}|{{\mathbf{n}}_{1:t - 1}}$ is Gaussian and thus the numerical integration techniques from section 4.1 can give accurate approximations of expected value terms for this density. Thus, we will overcome the challenge of incorporating Poisson observations and finding the terms in (13) by providing a novel set of derivations to convert the terms in (13) to expectations with respect to ${{\mathbf{x}}_t}|{{\mathbf{n}}_{1:t - 1}}$, thus allowing us to use the numerical integration tool.

Explicitly, the goal with these derivations is to convert each term in (13) to an expected value with respect to the Gaussian density of ${{\mathbf{x}}_t}|{{\mathbf{n}}_{1:t - 1}}$. We define the following notation for the likelihood mean and variance of ${{\mathbf{n}}_t}|{{\mathbf{x}}_t}$, which we emphasize are functions of ${{\mathbf{x}}_t}$:

\begin{align*}\begin{aligned} p\left( {{{\mathbf{x}}_t}} \right) \triangleq E\left[ {{{\mathbf{n}}_t}{\text{|}}{{\mathbf{x}}_t}} \right] \hfill \\ q\left( {{{\mathbf{x}}_t}} \right) \triangleq V\left[ {{{\mathbf{n}}_t}{\text{|}}{{\mathbf{x}}_t}} \right] \hfill \\ \end{aligned} .\end{align*}

For Poisson observations, these are given in (10). We will use the law of total expectation and the law of total variance [86] which for random variables $X$ and $Y$ state that ${E_Y}\left[ Y \right] = {E_X}\left[ {E\left[ {Y{\text{|}}X} \right]} \right]$ and ${V_Y}\left[ Y \right] = {E_X}\left[ {V\left[ {Y{\text{|}}X} \right]} \right] + {V_X}\left[ {E\left[ {Y{\text{|}}X} \right]} \right]$, respectively. We start with deriving the first term in (13) by using the law of total expectation followed by conditional independence:

\begin{align*}\begin{aligned} {\hat {\mathbf{n}}_{t|t - 1}} &amp; = {E_{\mathbf{n}}}\left[ {{{\mathbf{n}}_t}{\text{|}}{{\mathbf{n}}_{1:t - 1}}} \right] \hfill \\ &amp; = {E_{\mathbf{x}}}\left[ {E\left[ {{{\mathbf{n}}_t}\left| {{{\mathbf{x}}_t},{{\mathbf{n}}_{1:t - 1}}} \right.} \right]{\text{|}}{{\mathbf{n}}_{1:t - 1}}} \right] \hfill \\ &amp; = {E_{\mathbf{x}}}\left[ {E\left[ {{{\mathbf{n}}_t}\left| {{{\mathbf{x}}_t}} \right.} \right]{\text{|}}{{\mathbf{n}}_{1:t - 1}}} \right] \hfill \\ &amp; = {E_{\mathbf{x}}}\left[ {p\left( {{{\mathbf{x}}_t}} \right){\text{|}}{{\mathbf{n}}_{1:t - 1}}} \right] \hfill \\ \end{aligned} .\end{align*}

We emphasize that while initially the expectation is taken with respect to ${{\mathbf{n}}_t}|{{\mathbf{n}}_{1:t - 1}}$ on the first line, the final line is written as an expectation with respect to the Gaussian ${{\mathbf{x}}_t}|{{\mathbf{n}}_{1:t - 1}}$ which is the goal of this derivation. To derive the second term in (13), we start with the law of total variance and conditional independence:

\begin{align*}\begin{aligned} {{\boldsymbol{\Lambda }}_{{\mathbf{nn}}}} &amp; = {V_{\mathbf{n}}}\left[ {{{\mathbf{n}}_t}{\text{|}}{{\mathbf{n}}_{1:t - 1}}} \right] \hfill \\ &amp; = {E_{\mathbf{x}}}\left[ {V\left[ {{{\mathbf{n}}_t}\left| {{{\mathbf{x}}_t}} \right.} \right]{\text{|}}{{\mathbf{n}}_{1:t - 1}}} \right] + {V_{\mathbf{x}}}\left[ {E\left[ {{{\mathbf{n}}_t}\left| {{{\mathbf{x}}_t}} \right.} \right]{\text{|}}{{\mathbf{n}}_{1:t - 1}}} \right] \hfill \\ &amp; = {E_{\mathbf{x}}}\left[ {q\left( {{{\mathbf{x}}_t}} \right){\text{|}}{{\mathbf{n}}_{1:t - 1}}} \right] + {V_{\mathbf{x}}}\left[ {p\left( {{{\mathbf{x}}_t}} \right){\text{|}}{{\mathbf{n}}_{1:t - 1}}} \right] \hfill \\ &amp; = {E_{\mathbf{x}}}\left[ {q\left( {{{\mathbf{x}}_t}} \right){\text{|}}{{\mathbf{n}}_{1:t - 1}}} \right] + {E_{\mathbf{x}}}\left[ {p\left( {{{\mathbf{x}}_t}} \right)p{{\left( {{{\mathbf{x}}_t}} \right)}^T}{\text{|}}{{\mathbf{n}}_{1:t - 1}}} \right]\\ &amp; \quad - {E_{\mathbf{x}}}\left[ {p\left( {{{\mathbf{x}}_t}} \right){\text{|}}{{\mathbf{n}}_{1:t - 1}}} \right]{E_{\mathbf{x}}}{\left[ {p\left( {{{\mathbf{x}}_t}} \right){\text{|}}{{\mathbf{n}}_{1:t - 1}}} \right]^T}\,\,\,\, \hfill \\ &amp; = {E_{\mathbf{x}}}\left[ {q\left( {{{\mathbf{x}}_t}} \right){\text{|}}{{\mathbf{n}}_{1:t - 1}}} \right] + {E_{\mathbf{x}}}\left[ {p\left( {{{\mathbf{x}}_t}} \right)p{{\left( {{{\mathbf{x}}_t}} \right)}^T}{\text{|}}{{\mathbf{n}}_{1:t - 1}}} \right]\\ &amp; \quad - {\hat {\mathbf{n}}_{t|t - 1}}{\hat {\mathbf{n}}_{t|t - 1}}{\,^T} \hfill \\ \end{aligned} .\end{align*}

The fourth line results from expanding ${V_{\mathbf{x}}}\left[ {p\left( {{{\mathbf{x}}_t}} \right){\text{|}}{{\mathbf{n}}_{1:t - 1}}} \right]$ using the definition of variance. Thus, the final line consists of ${\hat {\mathbf{n}}_{t|t - 1}}$ which is found in (15) and two expected values with respect to the Gaussian ${{\mathbf{x}}_t}|{{\mathbf{n}}_{1:t - 1}}$, thus satisfying the goal for this term. To derive the last term in (13), we show a full proof in appendix section A.2. based on the law of total expectation, which gives the following:

\begin{align*}\begin{aligned} {{\boldsymbol{\Lambda }}_{{\mathbf{xn}}}} &amp; = {E_{{\mathbf{x}},{\mathbf{n}}}}\left[ {\left( {{{\mathbf{x}}_t} - {{\hat {\mathbf{x}}}_{t|t - 1}}} \right){{\left( {{{\mathbf{n}}_t} - {{\hat {\mathbf{n}}}_{t|t - 1}}} \right)}^T}\left| {{{\mathbf{n}}_{1:t - 1}}} \right.} \right]\,\, \hfill \\ &amp; = {E_{{\mathbf{x}},{\mathbf{n}}}}\left[ {{{\mathbf{x}}_t}{\mathbf{n}}_t^T\left| {{{\mathbf{n}}_{1:t - 1}}} \right.} \right] - {\hat {\mathbf{x}}_{t|t - 1}}\hat {\mathbf{n}}_{t|t - 1}^{\text{T}}\,\, \hfill \\ &amp; = {E_{\mathbf{x}}}\left[ {{{\mathbf{x}}_t}E{{\left[ {{{\mathbf{n}}_t}|{{\mathbf{x}}_t}} \right]}^T}\left| {{{\mathbf{n}}_{1:t - 1}}} \right.} \right] - {\hat {\mathbf{x}}_{t|t - 1}}\hat {\mathbf{n}}_{t|t - 1}^{\text{T}} \hfill \\ &amp; = {E_{\mathbf{x}}}\left[ {{{\mathbf{x}}_t}p{{\left( {{{\mathbf{x}}_t}} \right)}^T}\left| {{{\mathbf{n}}_{1:t - 1}}} \right.} \right] - {\hat {\mathbf{x}}_{t|t - 1}}\hat {\mathbf{n}}_{t|t - 1}^{\text{T}} \hfill \\ \end{aligned} .\end{align*}

The final line is an expected value with respect to the Gaussian ${{\mathbf{x}}_t}|{{\mathbf{n}}_{1:t - 1}}$, satisfying the goal of the derivation for the last term. Together, (15)–(17) give the final equations to be approximated with the numerical integration tool in 5.1. We emphasize that these equations are analytically derived and require only the likelihood mean and covariance of the observation modality as in (10), which we know for a Poisson observation model.

#### 4.3.1. Completed PCF

We now combine the numerical integration tool of 5.1 with the newly derived expected value terms of (15)–(17) that are now all with respect to a Gaussian to yield the full PCF. The approximate terms for (13) are then as follows:

\begin{align*}\begin{array}{l} {{\mathbf{x}}_i} \buildrel \Delta \over = {\hat {\mathbf{x}}_{t|t - 1}} + \sqrt {\hat {\boldsymbol{\Lambda }}}_{t\left| {t - 1} \right.}{{{\boldsymbol{\unicode{x03BE}}}_i}}\\ {{{\hat {\mathbf{n}}}}_{t\left| {t - 1} \right.}} \approx \sum\limits_{i = 1}^{2{d^2} + 1} {{w_i}} \cdot p\left( {{{\mathbf{x}}_i}} \right)\\ {{\boldsymbol{\Lambda }}_{{\mathbf{nn}}}} \approx \sum\limits_{i = 1}^{2{d^2} + 1} {{w_i}} \! \cdot \!\left(\! {q\left( {{{\mathbf{x}}_i}} \right) + p\left( {{{\mathbf{x}}_i}} \right)p{{\left( {{{\mathbf{x}}_i}} \right)}^T}} \!\right)\! - \!{\hat {\mathbf{n}}_{t|t - 1}}\hat {\mathbf{n}}_{t|t - 1}^T\\ {{\boldsymbol{\Lambda }}_{{\mathbf{xn}}}} \approx \sum\limits_{i = 1}^{2{d^2} + 1} {{w_i}} \cdot {{\mathbf{x}}_i}p{\left( {{{\mathbf{x}}_i}} \right)^T} - {\hat {\mathbf{x}}_{t|t - 1}}\hat {\mathbf{n}}_{t|t - 1}^T \end{array} \end{align*}

with ${w_i}$ and ${{\boldsymbol{\xi }}_i}$ given in (6) and the likelihood mean and covariance of Poisson $p\left( \cdot \right)$ and $q\left( \cdot \right)$ given in (10). Thus, the new filter PCF consists of a prediction step given by the equations in (11) followed by the update step which incorporates the next observation ${{\mathbf{n}}_t}$ using the equations in (12) with terms approximated here in (18) with deterministically sampled points given in (6). We emphasize that prior non-linear filters that use numerical integration [37–40] are formulated for non-linear observations with the specific form of ${{\mathbf{n}}_t} = h\left( {{{\mathbf{x}}_t}} \right) + {{\mathbf{v}}_t}$ where ${{\mathbf{v}}_t}$ is additive noise with covariance ${\mathbf{R}}$. This form is distinct from the Poisson observation form of (8) which does not have an $h\left( \cdot \right)$ function nor additive noise with an ${\mathbf{R}}$ term. Thus, neither the equations nor the derivations from these prior works could be used for Poisson observations, and we needed to derive a new filter, which led to PCF. Thus, one contribution here is in the novel derivations of (15)–(17) that have not been shown by prior works and show how to more generally expand the terms in (13) through analytical techniques to be able to accommodate more general non-linear observations that do not take the specific form of ${{\mathbf{n}}_t} = h\left( {{{\mathbf{x}}_t}} \right) + {{\mathbf{v}}_t}$. A high-level overview of PCF is visualized in figures 1(a) and (b) with additional implementation details given in appendix section A.3. Note that PCF is a causal filter.

Unlike the new PCF, prior inference methods for Poisson observations often rely on the distinct method of Laplace approximation, leading to the widely-used causal PPF [13, 16, 17, 24]. Unlike PCF, Laplace estimates the mean and covariance based on a single point in the latent space while PCF’s deterministic sampling samples multiple points from the latent space to help improve accuracy. A trade-off for this sampling is that PCF incurs additional runtime which scales on the order of the number of sampled points, in this case ${d^2}$. Both PCF and PPF are causal and readily fit with the RTS smoother [2, 26, 30] to give non-causal estimates. This is important because non-causal smoothed estimated are necessary for unsupervised learning.

PCF was derived in a way to also be extendable to other observation modalities provided that the likelihood mean and covariance $E[{{\mathbf{n}}_t}|{{\mathbf{x}}_t}]$ and $V\left[ {{{\mathbf{n}}_t}{\text{|}}{{\mathbf{x}}_t}} \right]$ are known and evaluable. Further, for future extensions to multimodal discrete-continuous data [1, 23, 46, 56, 82, 87], PCF enables an information form [40] that we derive in appendix section A.3.

### 4.4. Unsupervised learning with EM

For unsupervised learning, we embed PCF within an EM framework [26, 27, 30, 74]. The goal and cost function with EM is to learn system parameters $\hat \theta $ which maximize the log-likelihood for a set of given training observations ${{\mathbf{n}}_{1:T}}$:

\begin{align*}\hat \theta = \arg \mathop {\max }\limits_\theta \log f\left( {{{\mathbf{n}}_{1:T}};\theta } \right).\end{align*}

EM is an iterative framework that alternates between an expectation step (E-step) and a maximization step (M-step) with theoretical guarantees to increase the log-likelihood in each iteration assuming no approximations. Assuming we are on iteration $k - 1$, the E-step involves using the current estimate of the model parameters ${\theta ^{\left( {k - 1} \right)}}$ to find expected values of the latent state given all observations (e.g. ${E_{\mathbf{x}}}[{{\mathbf{x}}_t}{\mathbf{x}}_{t - 1}^T|{{\mathbf{n}}_{1:T}}]$). The M-step involves using the expected value terms to update the model parameters and obtain their $k$th estimate by maximizing the expected complete data likelihood:

\begin{align*}{\theta ^{\left( k \right)}} = \arg \mathop {\max }\limits_{{\theta ^*}} {E_{\mathbf{x}}}\left[ {\log f\left( {{{\mathbf{x}}_{0:T}},{{\mathbf{n}}_{1:T}};{\theta ^*}} \right)\left| {{{\mathbf{n}}_{1:T}};{\theta ^{\left( {k - 1} \right)}}} \right.} \right].\end{align*}

This can be shown to increase the overall log-likelihood of (19). A combination of analytical and numerical methods can be used to solve this maximization. We provide all equations in appendix section A.4. For example, the dynamics matrix ${{\mathbf{A}}^*}$ that maximizes its contribution to (20) can be found as:

\begin{align*}{{\mathbf{A}}^*} &amp; = \left( {\mathop \sum \limits_{t = 1}^T {E_{\mathbf{x}}}\left[{{\mathbf{x}}_t}{\mathbf{x}}_{t - 1}^T\left| {{{\mathbf{n}}_{1:T}}} \right.\right]} \right)\nonumber\\ &amp; \quad \times {\left( {\mathop \sum \limits_{t = 1}^T {E_{\mathbf{x}}}\left[{{\mathbf{x}}_{t - 1}}{\mathbf{x}}_{t - 1}^T\left| {{{\mathbf{n}}_{1:T}}} \right.\right]} \right)^{ - 1}}.\end{align*}

For some other parameters, numerical methods must be used to maximize their contribution to (20).

We call EM a framework because different inference methods can be used in the E-step to yield the necessary terms for the M-step. Thus, both PCF and PPF [22–24] can be used, and once combined with the RTS smoother, give the complete E-step for EM. We use PCF-EM to indicate an EM learning method which uses PCF and PPF-EM to similarly indicate that using PPF. Comparison of the PCF-EM and PPF-EM then shows the benefit of the novel PCF for unsupervised learning as the filters are the only distinct elements within the EM learning framework.

An alternative to this filtering-smoothing (forward-backward) approach is a global approach as detailed in [12, 14, 25]. Here, a Laplace approximation is also used but applied over all available times to $f({{\mathbf{x}}_{1:T}}|{{\mathbf{n}}_{1:T}};\theta )$ to yield an approximate Gaussian distribution for it. Necessary terms for M-step can then be extracted from the mean and covariance of this Gaussian or alternatively through samples drawn from the Gaussian as done in [3] and here for our comparisons. Thus, this global Laplace method is non-causal and makes up the entirety of the E-step in these methods. We use LEM to indicate an EM method with global Laplace as the E-step.

### 4.5. Extending to switching dynamical systems

We now extend the developed PCF to switching dynamical systems as overviewed by figure 1(d).

#### 4.5.1. Model for switching dynamical systems

We start with the model for a switching dynamical system with Poisson observations. The key difference with the stationary model presented in equations (7)–(9) is the addition of what we call a regime state ${s_t}$ which is a first order Markov chain with $M$ possible regimes that dictates the parameters to use throughout the remainder of the model. Explicitly, we write this as:

\begin{align*}\begin{array}{*{20}{l}} {P\left( {s_t^{\left(\, j \right)}|s_{t - 1}^{\left( i \right)}} \right) = {{{\Phi }}_{\boldsymbol{j,i}}}} \\ {{{\mathbf{x}}_t} = {\mathbf{A}}\left( {{s_t}} \right){{\mathbf{x}}_{t - 1}} + {{\mathbf{w}}_t},{{\mathbf{w}}_t} \sim N\left( {\mathbf{0},{\mathbf{Q}}\left( {{s_t}} \right)} \right)} \\ {P\left( {{{\mathbf{n}}_t}|{{\mathbf{x}}_t},{s_t}} \right) = \mathop \prod \limits_{i = 1}^C \frac{{{{\left( {{\lambda _i}\left( {{{\mathbf{x}}_t},{s_t}} \right){{\Delta }}} \right)}^{n_t^i}}\exp \left( { - {\lambda _i}\left( {{{\mathbf{x}}_t},{s_t}} \right){{\Delta }}} \right)}}{{n_t^i!}}{\text{ }}} \\ {{\lambda _i}\left( {{{\mathbf{x}}_t},{s_t}} \right){{\Delta }} = \exp \left( {{\alpha _i}\left( {{s_t}} \right) + {{\boldsymbol{\beta }}_i}{{\left( {{s_t}} \right)}^T}{{\mathbf{x}}_t}} \right)} \end{array}\end{align*}

where ${\boldsymbol{\Phi }}$ is the regime transition matrix, $s_t^{\left(\, j \right)}$ and $s_{t - 1}^{\left( i \right)}$ respectively denote ${s_t} = j$ and ${s_{t - 1}} = i$, and the initial distribution of the regime state ${s_1}$ is denoted as $\pi $. The dynamics matrix ${\mathbf{A}}\left( {{s_t}} \right)$ can take on one of $M$ possibilities $\{ {{{\mathbf{A}}^{\left( 1 \right)}}, \ldots ,{{\mathbf{A}}^{\left( M \right)}}} \}$ where ${\mathbf{A}}( {s_t^{\left(\, j \right)}} ) = {{\mathbf{A}}^{(\, j )}}$. The same notation holds for the remaining model parameters. We also assume that the spiking activity is conditionally independent given both the latent state and the regime state.

#### 4.5.2. Overview of switching filter and switching smoother

Our recently developed switching filter [1] for multiscale observations used the Laplace’s approximation. Here, we show how instead of Laplace’s method, we can use PCF to enable accurate inference for switching dynamical systems with Poisson observations.

The goal with causal filtering for switching systems is to calculate both the expected value of the latent state $E[{{\mathbf{x}}_t}|{{\mathbf{n}}_{1:t}}]$ and the probability of the regime state $P\left( {{s_t}{\text{|}}{{\mathbf{n}}_{1:t}}} \right)$ given the observations up to the present time ${{\mathbf{n}}_{1:t}}$. As one of the primary steps of the switching filter, it can be shown that after finding the mean and covariance of an intermediate distribution $f\left( {{{\mathbf{x}}_{t - 1}}{\text{|}}{{\mathbf{n}}_{1:t - 1}},s_t^{\left(\, j \right)}} \right)$, a stationary filter using parameters associated with regime $j$ is needed to incorporate the new observation ${{\mathbf{n}}_t}$ to find the mean and covariance of $f\left( {{{\mathbf{x}}_t}{\text{|}}{{\mathbf{n}}_{1:t}},s_t^{\left(\, j \right)}} \right)$ denoted as $\hat {\mathbf{x}}_{t|t}^{\left(\, j \right)}$ and $\hat {\boldsymbol{\Lambda }}_{t|t}^{\left(\, j \right)}$. In [1], to get the stationary filter we use the Laplace approximation and thus a PPF for Poisson observations. Here, we show that instead we can embed the new PCF. This is repeated for each regime $j \in \left[ {1,M} \right]$, resulting in a bank of $M$ PCF’s being used with one for each regime.

In [1], we also derived a switching smoother to yield non-causal estimates of the latent and regime states. This smoother only requires the outputs of the switching filter and thus can directly be applied as presented in [1]. Compared with prior switching smoothers [33, 34], the new smoother was shown to be more robust and generalizable. For the purpose of unsupervised learning, additional expected value terms not directly calculated by the smoother are needed like $E[{{\mathbf{x}}_t}{{\mathbf{x}}_{t - 1}}|{{\mathbf{n}}_{1:T}},s_t^{\left(\, j \right)}]$. For these terms, we run additional RTS smoothers from the $f ( {{{\mathbf{x}}_t}{\text{|}}{{\mathbf{n}}_{1:T}},s_t^{\left(\, j \right)}} )$ densities to directly yield $f ( {{{\mathbf{x}}_{t - 1}}{\text{|}}{{\mathbf{n}}_{1:T}},s_t^{\left(\, j \right)}} )$ with equations in appendix section A.5.

#### 4.5.3. Unsupervised learning with switch EM framework

We now present the unsupervised learning method for switching dynamical systems with Poisson observations. Similar to stationary systems, we use an EM framework with the goal to learn system parameters $\hat \theta $ that maximize the log-likelihood of a given set of training observations ${{\mathbf{n}}_{1:T}}$:

\begin{align*}\hat \theta = \arg \mathop {\max }\limits_\theta \log f\left( {{{\mathbf{n}}_{1:T}};\theta } \right).\end{align*}

Here, only Poisson observations are available with both latent states and regime states being unobserved. Thus, this is a method to learn system parameters and subsequently identify regimes in data that maximize the log-likelihood. The E-step involves finding expected value terms within the expected complete data likelihood using the current estimate of model parameters. We define the following notation:

\begin{align*}\begin{aligned} E\left[ { \cdot |{{\mathbf{n}}_{1:T}};{\theta ^{\left( {k - 1} \right)}}} \right] &amp; \triangleq \langle \cdot \rangle \hfill \\ E\left[ { \cdot |{{\mathbf{n}}_{1:T}},s_t^{\left(\, j \right)};{\theta ^{\left( {k - 1} \right)}}} \right] &amp; \triangleq {\langle \cdot \rangle ^{\left(\, j \right)}} \hfill \\ \end{aligned} .\end{align*}

The expected complete data likelihood is then:

\begin{align*}\begin{aligned} &amp; {E_{{\mathbf{x}},s}}\left[ {\log f\left( {{{\mathbf{x}}_{0:T}},{s_{1:T}},{{\mathbf{n}}_{1:T}};{\theta ^*}} \right)\left| {{{\mathbf{n}}_{1:T}};{\theta ^{\left( {k - 1} \right)}}} \right.} \right] \hfill \\ &amp; \quad = {{\Sigma }}_{j = 1}^MP\left( {s_1^{\left(\, j \right)}{\text{|}}{{\mathbf{n}}_{1:T}}} \right)\log {\pi _j} \hfill \\ &amp; \qquad + {{\Sigma }}_{t = 1}^T{{\Sigma }}_{i,j = 1}^MP\left( {s_t^{\left(\, j \right)},s_{t - 1}^{\left( i \right)}{\text{|}}{{\mathbf{n}}_{1:T}}} \right)\log {{{\Phi }}_{j,i}} \hfill \\ &amp; \qquad + \langle \log N\left( {{{\mathbf{x}}_0};{{\boldsymbol{\unicode{x03BC}}}_0},{{\boldsymbol{\Lambda }}_0}} \right)\rangle \hfill \\ &amp; \qquad + {{\Sigma }}_{t = 1}^T{{\Sigma }}_{j = 1}^MP\left( {s_t^{\left(\, j \right)}{\text{|}}{{\mathbf{n}}_{1:T}}} \right) \hfill \\ &amp; \qquad \times {\left\langle \log N\left( {{{\mathbf{x}}_t};{{\mathbf{A}}^{\left(\, j \right)}}{{\mathbf{x}}_{t - 1}},{{\mathbf{Q}}^{\left(\, j \right)}}} \right)\right\rangle ^{\left(\, j \right)}} \hfill \\ &amp; \qquad + \,{{\Sigma }}_{t = 1}^T{{\Sigma }}_{j = 1}^MP\left( {s_t^{\left(\, j \right)}{\text{|}}{{\mathbf{n}}_{1:T}}} \right) \hfill \\ &amp; \qquad \times \left( {{{\Sigma }}_{i = 1}^C\log {\text{Poisson}}(n_t^i;\exp {{\left( {\alpha _i^{\left(\, j \right)} + {\boldsymbol{\beta }}{{_i^{\left(\, j \right)}}^T}{{\mathbf{x}}_t}} \right)}^{\left(\, j \right)}}} \right). \hfill \\ \end{aligned}\end{align*}

The M-step then finds parameters that maximize (25):

\begin{align*}{\theta ^{\left( k \right)}} &amp; = \arg \mathop {\max }\limits_{{\theta ^*}} {E_{{\mathbf{x}},s}}\nonumber\\ &amp; \quad \times \left[ {\log f\left( {{{\mathbf{x}}_{0:T}},{s_{1:T}},{{\mathbf{n}}_{1:T}};{\theta ^*}} \right)\left| {{{\mathbf{n}}_{1:T}};{\theta ^{\left( {k - 1} \right)}}} \right.} \right].\end{align*}

For example, the dynamics matrix ${{\mathbf{A}}^{\left(\, j \right)}}$ for regime $j$ that maximizes its contribution to (26) is:

\begin{align*}{{\mathbf{A}}^{\left(\, j \right)}}^* &amp; = \left( {\mathop \sum \limits_{t = 1}^T P\left( {s_t^{\left(\, j \right)}{\text{|}}{{\mathbf{n}}_{1:T}}} \right){E_{\mathbf{x}}}\left[ {{{\mathbf{x}}_t}{\mathbf{x}}_{t - 1}^T|{{\mathbf{n}}_{1:T}},s_t^{\left(\, j \right)}} \right]} \right)\nonumber\\ &amp; \quad \times {\left( {\mathop \sum \limits_{t = 1}^T P\left( {s_t^{\left(\, j \right)}{\text{|}}{{\mathbf{n}}_{1:T}}} \right){E_{\mathbf{x}}}\left[ {{{\mathbf{x}}_{t - 1}}{\mathbf{x}}_{t - 1}^T|{{\mathbf{n}}_{1:T}},s_t^{\left(\, j \right)}} \right]} \right)^{ - 1}}.\end{align*}

We provide all equations in appendix section A.4.

The main challenge with unsupervised learning for switching dynamical system is that finding expected value terms for the E-step exactly is intractable given that the number of regime combinations over time grows exponentially with time. To address this, we instead find approximate estimates of the expected value terms by running a switching filter with PCF embedded followed by a switching smoother as shown in the previous section. We refer to this switch EM framework with PCF as sPCF-EM. While not done in prior work, a PPF can also be embedded in the switch EM framework, which we refer to as sPPF-EM. Since the only difference between sPCF-EM and sPPF-EM is the novel PCF, we implement the sPPF-EM here and use these comparisons to show the critical need for PCF in unsupervised learning of switching dynamical systems.

To learn switching dynamical systems with Poisson observations, prior studies use a variational EM framework [3, 28, 29] instead of using switching filters and smoothers for the EM which we do here. Variational EM is closely related to EM in that it is an unsupervised learning method that finds model parameters with alternating expectation and maximization steps. However, the cost function that variational EM maximizes is the lower bound to the log-likelihood of the observations rather than the log-likelihood itself. This is achieved by taking expectations with respect to a simpler distribution through a mean-field variational approximation [29]. Prior work has developed a method called variational Laplace EM (vLEM) [3] that can be applied to the switching dynamical systems of this work, where the simpler distribution assumes the regime and latent states are independent of each other. This allows global Laplace to be used in its expectation step to find expected values of the latent state. While there are differences in the approach to finding expected values between sPCF-EM and vLEM, both result in estimated parameters of the same model which we focus our comparisons on.

### 4.6. Numerical simulation framework

We use extensive numerical simulations to validate the PCF-based EM frameworks. We simulate spiking activity from both stationary and switching linear dynamical systems with Poisson observations at various data lengths for training. For stationary systems, we learn models using PCF-EM, PPF-EM, and LEM. For switching systems, we learn models using sPCF-EM, sPPF-EM, and vLEM. We also separately simulate testing data for evaluating learned model parameters.

#### 4.6.1. Numerical simulation settings

For stationary systems, we randomly form 60 general systems with a time bin ${{\Delta }}$ of 2 ms and sweep the training sample size across 10 k, 31.6 k, 100 k, and 316 k samples, equivalent to 20 s at the shortest and 632 s at the longest. We separately simulate testing data per system at 10 k samples (20 s) of length that all learned models per system are evaluated on. To investigate how changes in time bin size affects learning, we also simulate 60 systems with a time bin ${{\Delta }}$ of 10 ms with sweeps of training data size of 6.32 k, 20 k, and 63.2 k samples corresponding to similar data durations as before. Separate test data is also simulated per system with 20 s of duration (2 k samples).

For the dynamics equation parameters, we set the dimension of ${{\mathbf{x}}_t}$: $\dim \left( {{{\mathbf{x}}_t}} \right) \triangleq d$ to 8 based on common values used in prior works [1, 20, 81] and randomly form the dynamics matrix ${\mathbf{A}}$ and noise covariance matrix ${\mathbf{Q}}$. Using the procedure described in [1, 23], we assume the eigenvalues of ${\mathbf{A}}$ are stable and comprised of $d/2$ complex-conjugate pairs ${\left\{ {{r_i}{e^{ \pm j{\theta _i}}}} \right\}_{i \in \left[ {1,d/2} \right]}}$ with ${r_i}$ dictating the signal decay time with half life ${t_{1/2}} = \ln 2\frac{{ - {{\Delta }}}}{{\ln {r_i}}}$ and ${\theta _i}$ dictating oscillation frequency given by $\frac{{{\theta _i}}}{{2\pi {{\Delta }}}}$. We randomly select eigenvalues with decay half-lives between [13, 277] ms (i.e. a range of [0.9, 0.995] for ${r_i}$ with ${{\Delta }} = 2$ ms) and frequencies between [0.8, 5] Hz (i.e. a range of [0.010, 0.063] for ${\theta _i}$ with ${{\Delta }} = 2$ ms) based on values seen in prior literature [76, 81]. We also randomly select the eigenvalues of ${\mathbf{Q}}$ to be between 0.01 and 0.04. We simulate latent states using (7). For the observation equation parameters, we assume the number of neurons to be 60 and randomly choose a base firing rate between 3 and 5 Hz and a maximum firing rate between 50 and 70 Hz as in [1]. We then simulate observations based on (8).

For switching systems, we also randomly form 60 general systems with the same time bin and sweep the same training sample sizes as for the stationary systems. We separately simulate testing data at 30 k samples of length. For the regime state settings, we set the number of regimes $M$ to be 3 and the dwell time to be 2 s for each regime. The dwell time is the average time spent within the same regime and can be calculated for regime $j$ by ${t_{{\text{dwell}}}} = \frac{{{\Delta }}}{{1 - {{{\Phi }}_{jj}}}}$ where ${\phi _{jj}}$ comes from (22). We also assume the probability of switching from regime $j$ to $i$ is equal for all $i \ne j$. For the dynamics equation parameters, we randomly form ${{\mathbf{Q}}^{\left(\, j \right)}}$ and the eigenvalues of ${{\mathbf{A}}^{\left(\, j \right)}}$ for each regime $j$. Following the procedure of [1], we also randomly select the eigenvectors of ${{\mathbf{A}}^{\left(\, j \right)}}$ with a transient control value of 2.4 such that transient effects in the latent state signal are bounded. For the observation equation parameters, we randomly select them for each regime $j$ following the same procedure for stationary systems. We simulate the data then based on (22).

#### 4.6.2. Learning and performance metrics

After simulating data, we then learn system parameters with all methods. We run each for 300 EM iterations to give each method sufficient time for convergence. We initialize parameters randomly for each system except for ${\mathbf{A}}$ which is set to $0.9*{\mathbf{I}}$. For switching systems, we also set initial ${{\boldsymbol{\Phi}}_{jj}}$’s to have dwell times of 4 s. For fair comparisons amongst learning methods, we run each for the same number of iterations and provide the same initial parameters per system.

Once parameters are learned, we evaluate the learning performance using separately generated test data. As we want this evaluation process to be blind to which method generated the learned parameters, in test data, we choose to focus on decoding metrics from the same PPF and switching-PPF decoders [1] for all learning methods. This means that once parameters are learned with any learning method, we use these already-learned parameters to construct the associated PPF or switching PPF and run these filters on test data. This choice also allows us to show that the benefits in PCF-based learning translate to benefits in real-time decoding applications even when Laplace-based decoders are used after model learning.

The first decoding metric that we calculate is the neural self-prediction metric which is the accuracy of one-step-ahead prediction of spiking activity ${{\mathbf{n}}_t}$ using past activity ${{\mathbf{n}}_{1:t - 1}}$ through the probability $P\left( {n_t^i \unicode{x2A7E} 1|{{\mathbf{n}}_{1:t - 1}}} \right)$. We use the probability $P\left( {n_t^i \unicode{x2A7E} 1|{{\mathbf{n}}_{1:t - 1}}} \right)$ per neuron to classify whether spikes were observed in time bin $t{\text{ }}$or not and obtain an area under the curve (AUC) measure. We then compute the average AUC over all neurons. We then normalize the average AUC to yield a metric that we call predictive power, which is ${\text{PP}} = 2*{\text{AUC}} - 1$ such that 0 is chance and 1 is perfect prediction. To calculate the above probability, we derive the following for stationary systems:

\begin{align*}\begin{aligned} &amp; P\left( {n_t^i \unicode{x2A7E} 1|{{\mathbf{n}}_{1:t - 1}}} \right)\\ &amp; \quad = 1 - P\left( {n_t^i = 0|{{\mathbf{n}}_{1:t - 1}}} \right) \hfill \\ &amp; \quad = 1 - \mathop \smallint \nolimits P\left( {n_t^i = 0|{{\mathbf{x}}_t},{{\mathbf{n}}_{1:t - 1}}} \right)f\left( {{{\mathbf{x}}_t}{\text{|}}{{\mathbf{n}}_{1:t - 1}}} \right)d{{\mathbf{x}}_t} \hfill \\ &amp; \quad = 1 - \mathop \smallint \nolimits P\left( {n_t^i = 0|{{\mathbf{x}}_t}} \right)f\left( {{{\mathbf{x}}_t}{\text{|}}{{\mathbf{n}}_{1:t - 1}}} \right)d{{\mathbf{x}}_t} \hfill \\ &amp; \quad = 1 - \mathop \smallint \nolimits \exp \left( { - {\lambda _i}\left( {{{\mathbf{x}}_t}} \right){{\Delta }}} \right)f\left( {{{\mathbf{x}}_t}{\text{|}}{{\mathbf{n}}_{1:t - 1}}} \right)d{{\mathbf{x}}_t} \hfill \\ &amp; \quad = 1 - {E_{\mathbf{x}}}\left[ {\exp \left( { - {\lambda _i}\left( {{{\mathbf{x}}_t}} \right){{\Delta }}} \right){\text{|}}{{\mathbf{n}}_{1:t - 1}}} \right] \hfill \\ \end{aligned} \end{align*}

where we marginalize over ${{\mathbf{x}}_t}$ in the second line and use conditional independence in the third line. While there is no closed form solution to the expectation in the fifth line, we can use the numerical integration tool from section 4.1 to yield an accurate approximation as the expectation is with respect to the Gaussian ${{\mathbf{x}}_t}|{{\mathbf{n}}_{1:t - 1}}$. For switching systems, we first marginalize over the regime state:

\begin{align*} \begin{aligned} &amp; P\left( {n_t^i \unicode{x2A7E} 1|{{\mathbf{n}}_{1:t - 1}}} \right) \\ &amp; \quad\qquad= {{\Sigma }}_{j = 1}^M{\text{ }}P\left( {n_t^i \unicode{x2A7E} 1|{{\mathbf{n}}_{1:t - 1}},s_t^{\left(\, j \right)}} \right)P\left( {s_t^{\left(\, j \right)}{\text{|}}{{\mathbf{n}}_{1:t - 1}}} \right) \end{aligned}\end{align*}

\begin{align*}\begin{aligned} &amp; P\left( {n_t^i \unicode{x2A7E} 1|{{\mathbf{n}}_{1:t - 1}},s_t^{\left(\, j \right)}} \right) \hfill \\ &amp; \quad\qquad = 1 - P\left( {n_t^i = 0|{{\mathbf{n}}_{1:t - 1}},s_t^{\left(\, j \right)}} \right) \hfill \\ &amp; \quad\qquad = 1 - {E_{\mathbf{x}}}\left[ {\exp \left( { - {\lambda _i}\left( {{{\mathbf{x}}_t},s_t^{\left(\, j \right)}} \right){{\Delta }}} \right)|{{\mathbf{n}}_{1:t - 1}},s_t^{\left(\, j \right)}} \right] \hfill \\ \end{aligned} .\end{align*}

The expectation in the 3rd line of (30) is then approximated with (6). In simulations where ground truth parameters are known, we further calculate the predictive power using the true parameters $P{P_{\text{true}}}$ to yield a normalized predictive power metric $PP/P{P_{\text{true}}}$ where 0 is chance and 1 is true performance. This gives an indicator for if the learned parameters can capture the data as well as the true parameters can.

For switching systems in simulation, we also evaluate the decoded regime states ${\hat s_t}$ which is taken as the regime with the highest probability ${\hat s_t} = \arg \mathop {\max }\limits_j P(s_t^{\left(\, j \right)}|{{\mathbf{n}}_{1:t}})$. We compute the regime accuracy, or *Acc* as the proportion of estimated regime states that match the true regime states:

\begin{align*}Acc\left( {{s_{1:T}},{{\hat s}_{1:T}}} \right) = \mathop \sum \limits_{t = 1}^T \left[ {{s_t} = {{\hat s}_t}} \right]/T\end{align*}

where $\left[ {{s_t} = {{\hat s}_t}} \right]$ is 1 if the values match and 0 otherwise. As the learning method is unsupervised, the indices of the learned regimes may be a permutation of the true regimes (e.g., the 1st learned regime is the 2nd true regime) and thus we calculate the accuracy for all permutations of $1:M$ and choose the maximum. We further find the accuracy using true parameters to calculate a normalized accuracy. Because chance level accuracy is $1/M$ for these simulated systems, we calculate normalized accuracy as $\left( {Acc - \frac{1}{M}} \right)/\left( {Ac{c_{\text{true}}} - \frac{1}{M}} \right)$ such that 0 is chance and 1 is true performance.

To evaluate decoded latent states in simulation, additional methods are required to first learn a similarity transform because the latent states are unobserved. This means that there exists an infinite number of equivalent state space models through a reversible similarity transform [20], preventing direct comparisons of the true latent states with the decoded latent states. Thus, we use the procedure in [20] to first separately generate an additional $q = 1000d$ samples with $d$ being the dimension of the latent states purely to find such a similarity transform. We then use both the true model parameter and learned model parameters to find predicted latent state time series $\hat {\mathbf{x}}_{t|t - 1}^{{\text{true}}}$ and $\hat {\mathbf{x}}_{t|t - 1}^{{\text{learn}}}$ respectively. We then find the similarity transform which minimizes the mean-squared error between the predicted time series:

\begin{align*}\begin{aligned} \hat {\mathbf{L}} &amp; = \mathop {{\text{argmin}}}\limits_{\mathbf{L}} \left( {\mathop \sum \limits_t^T \left| {{\mathbf{L}}\hat {\mathbf{x}}_{t|t - 1}^{{\text{learn}}} - \hat {\mathbf{x}}_{t|t - 1}^{{\text{true}}}} \right|_2^2} \right) \hfill \\ &amp; = {\hat {\mathbf{X}}^{{\text{true}}}}{\hat {\mathbf{X}}^{{\text{learn}} \dagger}} \hfill \\ \end{aligned} \end{align*}

where ${\hat {\mathbf{X}}^{{\text{true}}}}$ and ${\hat {\mathbf{X}}^{{\text{learn}}}}$ are matrices whose columns are comprised of $\hat {\mathbf{x}}_{t|t - 1}^{{\text{true}}}$ and $\hat {\mathbf{x}}_{t|t - 1}^{{\text{learn}}}$ respectively. We then apply the similarity transform to the decoded latent states from the test set and evaluate the accuracy of the decoding using the average CC metric between the decoded and true latent states ($\hat {\mathbf{L}}{\hat {\mathbf{x}}_{t|t}}$ and ${{\mathbf{x}}_t}$) for each dimension, and then averaging over dimensions. We further find the average CC using true model parameters, $C{C_{\text{true}}}$, and calculate a normalized CC metric $CC/C{C_{\text{true}}}$ such that 0 is chance and 1 is true performance. This metric is found for both stationary and switching systems with the predicted time series for switching systems found by ${\hat {\mathbf{x}}_{t|t - 1}} = {{\Sigma }}_{j = 1}^M\hat {\mathbf{x}}_{t|t - 1}^{\left(\, j \right)}P(s_t^{\left(\, j \right)}|{{\mathbf{n}}_{1:t - 1}})$ with individual terms presented in [1].

For stationary systems, we further use the above similarity transform to directly evaluate the learned model parameters with respect to the true parameters. We apply the similarity transform to the learned parameters through a change of variables and quantify the error for each model parameter ${\boldsymbol{\Psi }}$ (for example, ${\mathbf{Q}}$) using the normalized matrix norm:

\begin{align*}{e_{{\Psi }}} = \frac{{{{\left| {{{{\Psi }}^{{\text{learn}}}} - {{{\Psi }}^{{\text{true}}}}} \right|}_F}}}{{{{\left| {{{{\Psi }}^{{\text{true}}}}} \right|}_F}}}\end{align*}

where ${\left| \cdot \right|_F}$ denotes the Frobenius norm. Further details can be found in [20]. We also quantify the normalized eigenvalue error:

\begin{align*}{e_{\text{eig}}} = \frac{{\mathop \sum \nolimits_{i = 1}^d \left| {{\text{eig}}_i^{{\text{learn}}} - {\text{eig}}_i^{{\text{true}}}} \right|}}{{\mathop \sum \nolimits_{i = 1}^d \left| {{\text{eig}}_i^{{\text{true}}}} \right|}}\end{align*}

where $\left| \cdot \right|$ for a complex number *a+bi* denotes the complex magnitude $\sqrt {{a^2} + {b^2}} $ and ${\text{ei}}{{\text{g}}_i}$ denotes the $i$th eigenvalue of dynamics matrix ${\mathbf{A}}$. We further match the learned eigenvalues to the true eigenvalues of ${\mathbf{A}}$ such that the normalized error is minimal. For switching systems, the learned regimes may not correspond to the true regimes. For example, if there is insufficient data, a method may learn a model that only uses one regime to describe all the data which could lead to misleading metrics if error is incorporated from misidentified regimes. Thus, we focus on decoding metrics that can show how well data is explained by a model even if regimes are not perfectly learned.

### 4.7. Experimental data

We further validate the developed methods using publicly available experimental data from the Sabes Lab [41] recorded from the primary motor cortex (M1) of a monkey (monkey I) performing a continuous series of reaches in a point-to-point task illustrated in figure 2. The task was structured such that circular targets were presented in an 8-by-8 square grid to the monkey in a virtual reality environment [41, 88]. The monkey was trained to make 2D reaches for targets via a cursor controlled by its fingertip on its left arm with target acquisition requiring the monkey to hold on the target within a set acceptance zone for 450 ms. Upon target acquisition, a new target would be randomly chosen and presented. Further details can be found in [41, 88]. We used a publicly available dataset from the Sabes Lab [41, 88], for which all animal procedures were performed in accordance with the U.S. National Research Council’s *Guide for the Care and Use of Laboratory Animals* and were approved by the UCSF Institutional Animal Care and Use Committee.

#### 4.7.1. Data preprocessing

Neural activity was acquired from a 96-channel silicon microelectrode array (Blackrock Microsystems, Salt Lake City, UT).

We randomly selected a subset of 30 channels for each recording day and used the multiunit spiking activity per channel as the neural signal comprised of the number of spikes per ${{\Delta }} = 10$ ms time bin. To investigate how changes in bin size affect learning similar to what was done in simulation, we also repeated this process but with the larger time bin size of ${{\Delta }} = 50$ ms. We analyzed data from the first five sessions in the month of 2016/09 (sessions 20160915_01–20160927_06) due to their consistent data lengths (38 k, 45 k, 36 k, 39 k, and 42 k samples respectively for ${{\Delta }} = 10$ ms).

#### 4.7.2. Learning procedure

We analyzed each session individually with a five-fold cross-validation scheme. Both stationary and switching learning methods were applied to each of the training sets comprised of four folds to yield learned model parameters. All learning methods applied were unsupervised and used only the spiking activity to generate the parameter estimates. To focus on switches in latent state dynamics, we set observation equation parameters to be the same across regimes. Decoded training set latent states were then linearly projected onto the 2D reach velocity which served as the behavior signal to learn a linear mapping from latent states to behavior. Based on section 2.3 that found that multiple initializations could improve consistency of results in simulation, 3 different parameter initializations were randomly generated such that each learning method was applied 3 times on a training set with these initial parameters. The final learned parameters for a given method was then chosen from the 3 based on whichever had the highest training set neural self-prediction.

#### 4.7.3. Evaluation pipeline

We then evaluate the learned parameters on the held-out test set data using decoding metrics similar to section 4.6.2. The procedure involved using the learned parameters with either PPF or switching PPF regardless of learning method. We first find the predictive power of the parameters based on the neural self-prediction metric. We then evaluate behavior decoding using the average CC metric by mapping the decoded latent states to 2D reach velocity using the projection learned in the training set. We finally interpret the decoded regime states with results found in section 2.5.

## Data Availability

No new data were created or analyzed in this study.

## Code availability statement

The code used to support the results will be available at https://github.com/ShanechiLab/sPCF-EM.

## Appendix

### A.1. Example cubature point set

We present an example point set from a 5th-degree spherical–radial cubature rule that would be used in (6). For Gaussian random variables ${\mathbf{X}}$ with dimension $d = 3$:

\begin{align*}\begin{aligned}{{\boldsymbol{\xi }}_1} &amp; = \left[ {\begin{array}{*{20}{c}} 0 \\ 0 \\ 0 \end{array}} \right] \hfill \\ {{\boldsymbol{\xi }}_{2:2d + 1}} &amp; = \sqrt {3 + 2} \cdot \left\{ \left[ {\begin{array}{*{20}{c}} 1 \\ 0 \\ 0 \end{array}} \right],\left[ {\begin{array}{*{20}{c}} { - 1} \\ 0 \\ 0 \end{array}} \right],\ldots ,\right.\\ &amp; \quad \left. \left[ {\begin{array}{*{20}{c}} 0 \\ 0 \\ 1 \end{array}} \right],\left[ {\begin{array}{*{20}{c}} 0 \\ 0 \\ { - 1} \end{array}} \right] \right\} \hfill \\ {{\boldsymbol{\xi }}_{2d + 2:2{d^2} + 1}} &amp;= \frac{{\sqrt {3 + 2} }}{{\sqrt 2 }} \cdot \left\{ \left[ {\begin{array}{*{20}{c}} 1 \\ 1 \\ 0 \end{array}} \right],\left[ {\begin{array}{*{20}{c}} 1 \\ { - 1} \\ 0 \end{array}} \right],\right.\\ &amp; \left. \quad \left[ {\begin{array}{*{20}{c}} { - 1} \\ 1 \\ 0 \end{array}} \right], \left[ {\begin{array}{*{20}{c}} { - 1} \\ { - 1} \\ 0 \end{array}} \right],\left[ {\begin{array}{*{20}{c}} 1 \\ 0 \\ 1 \end{array}} \right], \ldots , \right.\\ &amp; \quad \left. \left[ {\begin{array}{*{20}{c}} 0 \\ { - 1} \\ 1 \end{array}} \right],\left[ {\begin{array}{*{20}{c}} 0 \\ { - 1} \\ { - 1} \end{array}} \right] \right\} \hfill \\ \end{aligned} .\end{align*}

To give some further context and background on our choice of a spherical–radial cubature rule, the two rules that have been used in prior non-linear filters are the GHQ rule [39] and the spherical–radial cubature rule as used in [37, 38]. Briefly, the GHQ approximates 1 dimensional Gaussians with a fixed number of points $m$; this results in a tensor product across all dimensions of a $d$ dimensional Gaussian, which leads to a total of ${m^d}$ points and weights, thus scaling exponentially with latent state dimension [39]. For better computational complexity, the spherical–radial rule first uses a change of variable such that the function is integrated over hyperspheres along a radial axis where the points are now chosen from the surface of the spheres [37, 38]. Our choice for PCF to use the 5th-degree spherical–radial cubature rule [38] means that we only need $2{d^2} + 1$ points, thus allowing for polynomial scaling and avoiding the exponential scaling of the GHQ rule.

### A.2. Derivation of cross-covariance term in PCF

We derive the cross-covariance term (17) used in PCF as follows:

\begin{align*}\begin{aligned} {{\boldsymbol{\Lambda }}_{{\mathbf{xn}}}} &amp; = {E_{{\mathbf{x}},{\mathbf{n}}}}\left[ {\left( {{{\mathbf{x}}_t} - {{\hat {\mathbf{x}}}_{t|t - 1}}} \right){{\left( {{{\mathbf{n}}_t} - {{\hat {\mathbf{n}}}_{t|t - 1}}} \right)}^T}\left| {{{\mathbf{n}}_{1:t - 1}}} \right.} \right] \hfill \\ &amp; = {E_{{\mathbf{x}},{\mathbf{n}}}}\left[ {{{\mathbf{x}}_t}{\mathbf{n}}_t^T\left| {{{\mathbf{n}}_{1:t - 1}}} \right.} \right] - {\hat {\mathbf{x}}_{t|t - 1}}\hat {\mathbf{n}}_{t|t - 1}^{\text{T}} \hfill \\ \end{aligned} .\end{align*}

where the first term can be expanded as:

\begin{align*} &amp; {E_{{\mathbf{x}},{\mathbf{n}}}}\left[ {{{\mathbf{x}}_t}{\mathbf{n}}_t^T\left| {{{\mathbf{n}}_{1:t - 1}}} \right.} \right] \nonumber\\ &amp; \quad = \mathop \int \nolimits_{{{\mathbf{x}}_t}} \mathop \sum \limits_{{{\mathbf{n}}_t}} {{\mathbf{x}}_t}{\mathbf{n}}_t^Tf\left( {{{\mathbf{x}}_t},{{\mathbf{n}}_t}{\text{|}}{{\mathbf{n}}_{1:t - 1}}} \right){\text{d}}{{\mathbf{x}}_t} \hfill \nonumber\\ &amp; \quad = \mathop \int \nolimits_{{{\mathbf{x}}_t}} {{\mathbf{x}}_t}\left[ {\mathop \sum \limits_{{{\mathbf{n}}_t}} {\mathbf{n}}_t^TP\left( {{{\mathbf{n}}_t}{\text{|}}{{\mathbf{x}}_t},{{\mathbf{n}}_{1:t - 1}}} \right)} \right]f\left( {{{\mathbf{x}}_t}{\text{|}}{{\mathbf{n}}_{1:t - 1}}} \right){\text{d}}{{\mathbf{x}}_t} \hfill \nonumber\\ &amp; \quad = \mathop \int \nolimits_{{{\mathbf{x}}_t}} {{\mathbf{x}}_t}\left[ {\mathop \sum \limits_{{{\mathbf{n}}_t}} {\mathbf{n}}_t^TP\left( {{{\mathbf{n}}_t}{\text{|}}{{\mathbf{x}}_t}} \right)} \right]f\left( {{{\mathbf{x}}_t}{\text{|}}{{\mathbf{n}}_{1:t - 1}}} \right){\text{d}}{{\mathbf{x}}_t} \hfill \nonumber\\ &amp; \quad = \mathop \int \nolimits_{{{\mathbf{x}}_t}} {{\mathbf{x}}_t}E{\left[ {{{\mathbf{n}}_t}|{{\mathbf{x}}_t}} \right]^T}f\left( {{{\mathbf{x}}_t}{\text{|}}{{\mathbf{n}}_{1:t - 1}}} \right){\text{d}}{{\mathbf{x}}_t} \hfill \nonumber\\[10pt] &amp; \quad = {E_{\mathbf{x}}}\left[ {{{\mathbf{x}}_t}E{{\left[ {{{\mathbf{n}}_t}|{{\mathbf{x}}_t}} \right]}^T}\left| {{{\mathbf{n}}_{1:t - 1}}} \right.} \right] \hfill .\end{align*}

### A.3. Implementation details and the information form of PCF

Our choice of a 5th degree spherical–radial cubature rule is motivated by its comparable accuracy and improved efficiency relative to a 3rd degree GHQ rule as shown in [38]. However, one side effect of this is that the weights of the 5th degree cubature rule includes negative values, which leads to the possibility of instability [38]. Thus, we include in our implementation a check that the final update covariance ${\hat {\boldsymbol{\Lambda }}_{t|t}}$ is positive semi-definite and use PPF in the rare instances it is not positive semi-definite due to instability.

We also present an equivalent information form of the PCF that is used for the results of this work based on the statistical linear error propagation method [40, 89, 90]. The information form is an equivalent form of filters and can be readily combined with information filters of separate observation modalities [2].

To start, after the prediction step to find ${\hat {\mathbf{x}}_{t|t - 1}}$ and ${\hat {\boldsymbol{\Lambda }}_{t|t - 1}}$, we use numerical integration to approximate the cross-covariance ${{\boldsymbol{\Lambda }}_{{\mathbf{xn}}}}$ as in (18). We then make the approximation that this cross-covariance is equal to the following:

\begin{align*} {{\boldsymbol{\Lambda }}_{{\mathbf{xn}}}} &amp; = {E_{{\mathbf{x}},{\mathbf{n}}}}\left[ {\left( {{{\mathbf{x}}_t} - {{\hat {\mathbf{x}}}_{t|t - 1}}} \right){{\left( {{{\mathbf{n}}_t} - {{\hat {\mathbf{n}}}_{t|t - 1}}} \right)}^T}\left| {{{\mathbf{n}}_{1:t - 1}}} \right.} \right] \hfill \nonumber\\ &amp; \approx {\hat {\boldsymbol{\Lambda }}_{t|t - 1}}{\widetilde {\mathbf{C}}^T} \hfill .\end{align*}

where $\widetilde {\mathbf{C}}$ comes from an approximately linear likelihood mean of the observation:

\begin{align*}E\left[ {{{\mathbf{n}}_t}{\text{|}}{{\mathbf{x}}_t}} \right] \approx \widetilde {\mathbf{C}}{{\mathbf{x}}_t}.\end{align*}

Thus, a $\widetilde {\mathbf{C}}$ that would yield the same cross-covariance found from numerical integration is as follows:

\begin{align*}\widetilde {\mathbf{C}} = {\left( {\hat {\boldsymbol{\Lambda }}_{t|t - 1}^{ - 1}{{\boldsymbol{\Lambda }}_{{\mathbf{xn}}}}} \right)^T}.\end{align*}

The covariance ${{\boldsymbol{\Lambda }}_{{\mathbf{nn}}}}$ using (39) then becomes:

\begin{align*} {{\boldsymbol{\Lambda }}_{{\mathbf{nn}}}} &amp; = {E_{\mathbf{x}}}\left[ {V\left[ {{{\mathbf{n}}_t}\left| {{{\mathbf{x}}_t}} \right.} \right]{\text{|}}{{\mathbf{n}}_{1:t - 1}}} \right] + {V_{\mathbf{x}}}\left[ {E\left[ {{{\mathbf{n}}_t}\left| {{{\mathbf{x}}_t}} \right.} \right]{\text{|}}{{\mathbf{n}}_{1:t - 1}}} \right] \nonumber\\ &amp; = {E_{\mathbf{x}}}\left[ {q\left( {{{\mathbf{x}}_t}} \right){\text{|}}{{\mathbf{n}}_{1:t - 1}}} \right] + \widetilde {\mathbf{C}}{\hat {\boldsymbol{\Lambda }}_{t|t - 1}}{\widetilde {\mathbf{C}}^T} \nonumber\\ &amp; \triangleq \widetilde {\mathbf{R}} + \widetilde {\mathbf{C}}{\hat {\boldsymbol{\Lambda }}_{t|t - 1}}{\widetilde {\mathbf{C}}^T} \end{align*}

where $\widetilde {\mathbf{R}}$ indicates ${E_{\mathbf{x}}}\left[ {q\left( {{{\mathbf{x}}_t}} \right){\text{|}}{{\mathbf{n}}_{1:t - 1}}} \right]$ which is found with numerical integration. We can then apply the matrix inversion lemma [30] to yield the information form of the covariance update:

\begin{align*}\begin{aligned} {\hat {\boldsymbol{\Lambda }}_{t|t}} &amp; = {\hat {\boldsymbol{\Lambda }}_{t|t - 1}} - {{\boldsymbol{\Lambda }}_{{\mathbf{xn}}}}{\boldsymbol{\Lambda }}_{{\mathbf{nn}}}^{ - 1}{\boldsymbol{\Lambda }}_{{\mathbf{xn}}}^T \hfill \\ &amp; = {\left( {\hat {\boldsymbol{\Lambda }}_{t|t - 1}^{ - 1} + {{\widetilde {\mathbf{C}}}^T}{{\widetilde {\mathbf{R}}}^{ - 1}}\widetilde {\mathbf{C}}} \right)^{ - 1}} \hfill \\ \end{aligned} .\end{align*}

The mean update is then:

\begin{align*}{\hat {\mathbf{x}}_{t|t}} = {\hat {\mathbf{x}}_{t|t - 1}} + {\hat {\boldsymbol{\Lambda }}_{t|t}}{\widetilde {\mathbf{C}}^T}{\widetilde {\mathbf{R}}^{ - 1}}\left( {{{\mathbf{n}}_t} - {{\hat {\mathbf{n}}}_{t|t - 1}}} \right).\end{align*}

### A.4. M-step equations

We present here the equations for the M-step for switching systems. As stationary systems can be considered a special case of switching systems with the number of regimes $M = 1$, the same equations can be used with $\langle s_t^{\left(\, j \right)}\rangle $ set to 1 and all ${{\text{ }}^{\left(\, j \right)}}$ notation ignored to get the stationary version.

\begin{align*}\begin{aligned} &amp; E\left[ { \cdot \left| {{{\mathbf{n}}_{1:T}}} \right.} \right] \triangleq \langle \cdot \rangle \hfill \\ &amp; E\left[ { \cdot \left| {{{\mathbf{n}}_{1:T}},s_t^{\left(\, j \right)}} \right.} \right] \triangleq {\langle \cdot \rangle ^{\left(\, j \right)}} \hfill \\ &amp; \big\langle s_t^{\left(\, j \right)}\rangle = P\left( {s_t^{\left(\, j \right)}{\text{|}}{{\mathbf{n}}_{1:T}}} \right) \hfill \\ &amp; {{\mathbf{A}}^{\left(\, j \right)}}^*\! = \!\left(\! {\mathop \sum \limits_{t = 1}^T \Big\langle s_t^{\left(\, j \right)}\Big\rangle \big\langle{{\mathbf{x}}_t}{\mathbf{x}}{{_{t - 1}^T}\big\rangle^{\left(\, j \right)}}} \!\!\right)\!\!{\left(\! {\mathop \sum \limits_{t = 1}^T \big\langle s_t^{\left(\, j \right)}\big\rangle \big\langle{{\mathbf{x}}_{t - 1}}{\mathbf{x}}{{_{t - 1}^T}\big\rangle^{\left(\, j \right)}}} \!\!\right)^{ - 1}} \hfill \\ &amp; {{\mathbf{Q}}^{\left(\, j \right)}}^* = \Bigg( \mathop \sum \limits_{t = 1}^T \big\langle s_t^{\left(\, j \right)}\big\rangle \left( \big\langle{{\mathbf{x}}_t}{\mathbf{x}}{{_t^T}\big\rangle^{\left(\, j \right)}} - {{\mathbf{A}}^{\left(\, j \right)}}^*\big\langle {{\mathbf{x}}_{t - 1}}{\mathbf{x}}{{_t^T}\big\rangle^{\left(\, j \right)}}\right.\\ &amp; \;\,\qquad \left. - \big\langle{{\mathbf{x}}_t}{\mathbf{x}}{{_{t - 1}^T}\big\rangle^{\left(\, j \right)}}{{\mathbf{A}}^{\left(\, j \right)}}^*{\,^T} + {{\mathbf{A}}^{\left(\, j \right)}}^*\big\langle{{\mathbf{x}}_{t - 1}}{\mathbf{x}}{{_{t - 1}^T}\big\rangle^{\left(\, j \right)}}{{\mathbf{A}}^{\left(\, j \right)}}^*{\,^T} \right) \Bigg)\\ &amp; \;\,\qquad \times {\left( {\mathop \sum \limits_{t = 1}^T \langle s_t^{\left(\, j \right)} \rangle } \right)^{ - 1}} \hfill \\ &amp; {\boldsymbol{\unicode{x03BC}}}_0^* = \big\langle{{\mathbf{x}}_0}\big\rangle,\,\,{\boldsymbol{\Lambda }}_0^* = \big\langle{{\mathbf{x}}_0}{\mathbf{x}}_0^T\big\rangle - \big\langle{{\mathbf{x}}_0}\big\rangle \big\langle{{\mathbf{x}}_0}\big\rangle^T\, \hfill \\ &amp; {{\Phi }}_{j,i}^* = \left( {\mathop \sum \limits_{t = 2}^T \big\langle s_{t - 1}^{\left( i \right)}s_t^{\left(\, j \right)}}\big\rangle \right){\left( {\mathop \sum \limits_{t = 1}^{T - 1} \big\langle s_t^{\left(\, j \right)}}\big\rangle \right)^{ - 1}} \hfill \\ &amp; \pi _j^* = \left\langle s_1^{\left(\, j \right)} \right\rangle \hfill \end{aligned} \!\!\!\!.\end{align*}

For the observation equation parameters, we use numerical trust-region methods [91] as implemented in MATLAB.

\begin{align*}\begin{aligned} z_i^{\left(\, j \right)} &amp; \triangleq \left[ {\begin{array}{*{20}{c}} {\alpha _i^{\left(\, j \right)}} \\ {{\boldsymbol{\unicode{x03B2}}}_i^{\left(\, j \right)}} \end{array}} \right] \hfill \\ z_i^{\left(\, j \right)*} &amp; = \mathop {{\text{argmax}}}\limits_{\left[ {{\alpha _i};{{\boldsymbol{\unicode{x03B2}}}_i}} \right]} \mathop \sum \limits_{t = 1}^T \big\langle s_t^{\left(\, j \right)}\big\rangle \big\langle n_t^i\left( {{\alpha _i} + {\boldsymbol{\unicode{x03B2}}}_i^T{{\mathbf{x}}_t}} \right) \\ &amp; \quad - \exp {\left( {{\alpha _i} + {\boldsymbol{\unicode{x03B2}}}_i^T{{\mathbf{x}}_t}} \right)\big\rangle^{\left(\, j \right)}} \hfill \\ &amp; = \mathop {{\text{argmax}}}\limits_{\left[ {{\alpha _i};{{\boldsymbol{\unicode{x03B2}}}_i}} \right]} \mathop \sum \limits_{t = 1}^T \langle s_t^{\left(\, j \right)}\big\rangle\Bigg( n_t^i\left( {{\alpha _i} + {\boldsymbol{\unicode{x03B2}}}_i^T\hat {\mathbf{x}}_{t|T}^{\left(\, j \right)}} \right) \\ &amp; \quad -\ \exp \left( {{\alpha _i} + {\boldsymbol{\unicode{x03B2}}}_i^T\hat {\mathbf{x}}_{t|T}^{\left(\, j \right)} + \frac{1}{2}{\boldsymbol{\unicode{x03B2}}}_i^T\hat {\boldsymbol{\Lambda }}_{t|T}^{\left(\, j \right)}{\boldsymbol{\unicode{x03B2}}}_i^T} \right) \Bigg) \hfill \\ \end{aligned} .\end{align*}

### A.5. Additional equations for switching smoother for M-step terms

We use additional RTS smoothers to calculate additional terms necessary for the M-step. Given the mean and covariance $\hat {\mathbf{x}}_{t|T}^{\left(\, j \right)}$ and $\hat {\boldsymbol{\Lambda }}_{t|T}^{\left(\, j \right)}$ of the density $f ( {{{\mathbf{x}}_t}{\text{|}}{{\mathbf{n}}_{1:T}},s_t^{\left(\, j \right)}} )$, we apply the RTS smoother:

\begin{align*} \hat {\mathbf{x}}_{t - 1|T}^{\left( { - ,j} \right)} &amp; = \hat {\mathbf{x}}_{t - 1|t - 1}^{\left( { - ,j} \right)} + {{\mathbf{J}}_{t - 1}}\left( {\hat {\mathbf{x}}_{t|T}^{\left(\, j \right)} - \hat {\mathbf{x}}_{t|t - 1}^{\left(\, j \right)}} \right) \nonumber\\ \hat {\boldsymbol{\Lambda }}_{t - 1|T}^{\left( { - ,j} \right)} &amp; = \hat {\boldsymbol{\Lambda }}_{t - 1|t - 1}^{\left( { - ,j} \right)} + {{\mathbf{J}}_{t - 1}}\left( {\hat {\boldsymbol{\Lambda }}_{t|T}^{\left(\, j \right)} - \hat {\boldsymbol{\Lambda }}_{t|t - 1}^{\left(\, j \right)}} \right){\mathbf{J}}_{t - 1}^T \nonumber\\ \hat {\boldsymbol{\Lambda }}_{t,t - 1|T}^{\left( { - ,j} \right)} &amp; = \hat {\boldsymbol{\Lambda }}_{t|T}^{\left(\, j \right)}{\mathbf{J}}_{t - 1}^T \nonumber\\ {{\mathbf{J}}_{t - 1}} &amp; = \hat {\boldsymbol{\Lambda }}_{t - 1|t - 1}^{\left( { - ,j} \right)}{{\mathbf{A}}^{\left(\, j \right)}}^T\hat {\boldsymbol{\Lambda }}_{t|t - 1}^{{{\left(\, j \right)}^{ - 1}}} \end{align*}

where $\hat {\mathbf{x}}_{t - 1|T}^{\left( { - ,j} \right)}$ and $\hat {\boldsymbol{\Lambda }}_{t - 1|T}^{\left( { - ,j} \right)}$ are the mean and covariance of $f ( {{{\mathbf{x}}_{t - 1}}{\text{|}}{{\mathbf{n}}_{1:T}},s_t^{\left(\, j \right)}} )$ and $\hat {\boldsymbol{\Lambda }}_{t,t - 1|T}^{\left( { - ,j} \right)}$ is the cross-covariance between ${{\mathbf{x}}_t}$ and ${{\mathbf{x}}_{t - 1}}$ given ${{\mathbf{n}}_{1:T}}$ and $s_t^{\left(\, j \right)}$. The remaining necessary terms are found in the switching filter of [1] with the same notation. The necessary terms for the M-step are then as follows:

\begin{align*}\begin{aligned} E\left[ {{{\mathbf{x}}_t}{\mathbf{x}}_{t - 1}^T{\text{|}}{{\mathbf{n}}_{1:T}},s_t^{\left(\, j \right)}} \right] &amp; = \hat {\boldsymbol{\Lambda }}_{t,t - 1|T}^{\left( { - ,j} \right)} + \hat {\mathbf{x}}_{t|T}^{\left(\, j \right)}\hat {\mathbf{x}}_{t - 1|T}^{{{\left( { - ,j} \right)}^T}} \hfill \\ E\left[ {{{\mathbf{x}}_{t - 1}}{\mathbf{x}}_{t - 1}^T{\text{|}}{{\mathbf{n}}_{1:T}},s_t^{\left(\, j \right)}} \right] &amp; = \hat {\boldsymbol{\Lambda }}_{t - 1|T}^{\left( { - ,j} \right)} + \hat {\mathbf{x}}_{t - 1|T}^{\left( { - ,j} \right)}\hat {\mathbf{x}}_{t - 1|T}^{{{\left( { - ,j} \right)}^T}} \hfill \\ \end{aligned} \end{align*}
